# Tranexamic acid use and progression to clinically important bleeding in critically ill patients with non-clinically important non-variceal upper gastrointestinal bleeding: a retrospective cohort study

**DOI:** 10.3389/fmed.2026.1940595

**Published:** 2026-10-01

**Authors:** Ji Luo, Zheng Lei, Yuanjun Zhang, Zhiwei Yao, Xin Fu, Luping Wang, Shucheng Zhao, Jing Yang, Bo Wang, Hao Yang, Qin Wu

**Affiliations:** 1 Department of Critical Care Medicine, West China Hospital of Sichuan University-Ziyang Hospital, Ziyang Central Hospital, Ziyang, Sichuan, China; 2 Department of Critical Care Medicine, West China Hospital, Sichuan University, Chengdu, Sichuan, China

**Keywords:** clinically important gastrointestinal bleeding, in-hospital mortality, non-clinically important nonvariceal upper gastrointestinal bleeding, thrombotic, tranexamic acid

## Abstract

Tranexamic acid (TXA) is widely used to reduce bleeding in perioperative and hemorrhagic conditions, but its role in upper gastrointestinal bleeding (UGIB) remains controversial. This single-center retrospective cohort study evaluated whether TXA use was associated with reduced progression to clinically important gastrointestinal bleeding (GIB) in critically ill patients with non-clinically important non-variceal UGIB. Adult ICU patients who developed non-clinically important non-variceal UGIB between 2018 and 2022 were included. Among them, 154 patients receiving TXA within 24 h after bleeding onset (mean duration 1.71 ± 1.42 days; mean total dose 2.12 ± 2.36 g) were compared with 1,660 patients who received neither TXA nor other hemostatic agents. After propensity score matching, 154 matched pairs were analyzed. Progression to clinically important GIB did not differ significantly between the TXA and non- TXA groups before matching (5.8% vs. 5.7%; *P*=0.951, 95% CI: 0.450–1.707) or after matching (5.8% vs. 7.8%; *P*=0.664, 95% CI: 0.562–3.164); Exploratory univariate conditional logistic regression analysis showed that TXA use was not associated with this outcome (OR 1.333, 95% CI 0.562–3.164; *P*=0.514). In the matched cohort, TXA was not associated with significant differences in endoscopy requirement, transfusion requirement, hospital length of stay, in-hospital mortality, or thrombotic complications. Inverse probability of treatment weighting analysis yielded consistent results for the primary outcome (5.1% vs. 5.8%; *P*=0.418, 95% CI: 0.840–1.522). In critically ill patients with non-clinically important non-variceal UGIB, TXA use was not associated with a statistically detectable reduction in progression to clinically important GIB or improvements in major clinical outcomes. Routine empirical use of TXA in this population should be approached with caution.

## 1 Introduction

Gastrointestinal bleeding remains a common acute-care presentation associated with substantial morbidity, mortality, and healthcare resource utilization (1). Research in this field has expanded considerably over recent decades (2). Upper gastrointestinal bleeding (UGIB) is a common complication among critically ill patients and is frequently related to stress-related mucosal injury, impaired mucosal perfusion, systemic inflammation, coagulation abnormalities, and the use of medications that interfere with hemostasis (3). In the intensive care unit (ICU), the clinical spectrum of UGIB is heterogeneous, ranging from mild manifestations such as coffee-ground gastric aspirate, hematemesis, or melena to clinically important bleeding characterized by hemodynamic instability, substantial hemoglobin decline, transfusion requirement, or the need for urgent endoscopic or surgical intervention (4). Although clinically important GIB has been widely recognized as a major adverse event, non-clinically important non-variceal UGIB is encountered more frequently in daily ICU practice and often creates uncertainty regarding optimal management (3, 5).

Tranexamic acid (TXA), a synthetic lysine analogue that inhibits plasminogen activation and suppresses fibrinolysis, has become an important pharmacological strategy for reducing bleeding in multiple clinical settings (6). In recent years, its role in perioperative blood management has received increasing attention. Large trials and meta-analyses have shown that TXA reduces perioperative blood loss and transfusion requirements across cardiac and orthopedic surgery without a clear signal of increased thrombotic risk in appropriately selected populations (7–10).

Beyond the perioperative setting, TXA may also have a role as an adjunctive hemostatic treatment in emergency department practice for selected mucosal bleeding syndromes, such as epistaxis (11). However, its effectiveness appears to be context-dependent and may vary according to the bleeding site, administration route, and comparator therapy.

The success of TXA in perioperative bleeding prevention and its potential utility in mucosal bleeding provide a strong biological and clinical rationale for considering its use in other bleeding-related conditions. In theory, TXA may be beneficial in upper gastrointestinal mucosal bleeding because local fibrinolytic activity may contribute to clot instability and recurrent bleeding at ulcer or erosion sites. By inhibiting plasmin-mediated fibrin degradation, TXA could help stabilize formed clots and reduce the risk of bleeding progression (12, 13). This mechanism is particularly attractive in ICU patients, in whom stress-related mucosal injury, inflammation, organ dysfunction, and coagulation disturbances may coexist.

However, the clinical value of TXA in gastrointestinal bleeding remains controversial. Unlike perioperative bleeding, which is often predictable, time-defined, and related to a controlled surgical insult, gastrointestinal bleeding is frequently heterogeneous in onset, aetiology, severity, and timing of recognition. Previous studies and meta-analyses have reported inconsistent effects of TXA on mortality, rebleeding, transfusion requirement, and need for intervention in UGIB (8, 14). Moreover, The largest trial to date in this field, the HALT-IT trial, randomized 12,009 patients with significant gastrointestinal bleeding and found no reduction in death due to bleeding within 5 days (4% vs. 4%; RR 0.99, 95% CI 0.82–1.18), with an increased risk of venous thromboembolic events (0.8% vs. 0.4%; RR 1.85, 95% CI 1.15–2.98). Importantly, HALT-IT primarily enrolled patients with established, clinically important bleeding, whereas the present study focuses on patients with initially non-clinically important bleeding, a population largely underrepresented in that trial. (15). As a result, current guidelines do not routinely recommend TXA for UGIB (16, 17). This contrast between the expanding perioperative use of TXA and the uncertainty surrounding its role in gastrointestinal bleeding highlights the need to define the specific clinical contexts in which TXA may or may not be useful.

This uncertainty is especially relevant in critically ill patients with non-clinically important non-variceal UGIB. These patients differ substantially from general UGIB populations and perioperative cohorts. They often have severe underlying illness, sepsis, shock, mechanical ventilation, renal or hepatic dysfunction, anticoagulant exposure, and a high baseline risk of both bleeding and thrombosis. In addition, non-clinically important bleeding episodes in the ICU are often self-limited and may not require endoscopic or surgical intervention. Nevertheless, clinicians may empirically administer TXA because of concern that minor bleeding could progress to clinically important haemorrhage. Whether such practice is associated with improved outcomes remains unclear.

Therefore, while TXA has an established role in perioperative blood management and selected mucosal bleeding conditions, its benefit-risk profile in critically ill patients with non-clinically important non-variceal UGIB remains uncertain. In this retrospective cohort study, we evaluated the association between TXA use and clinical outcomes in ICU patients who developed non-clinically important non-variceal UGIB.

## 2 Methods

### 2.1 Study design and setting

This retrospective cohort study was conducted in the Department of Critical Care Medicine, West China Hospital, Sichuan University, China. We reviewed the electronic medical records of all adult patients admitted to the intensive care unit between January 2018 and December 2022. The study was approved by the institutional ethics committee of West China Hospital, Sichuan University. The requirement for informed consent was waived because of the retrospective nature of the study and the use of de-identified clinical data (Ethics No 2021S673).

### 2.2 Study population

Adult patients admitted to the intensive care unit (ICU) were eligible for inclusion if they met all of the following criteria: (1) age ≥18 years; (2) ICU length of stay ≥48 h; and (3) development of non-clinically important non-variceal upper gastrointestinal bleeding (UGIB) during ICU stay, with the bleeding episode occurring at least 48 h after ICU admission. Patients were excluded if they met any of the following criteria: (1) clinically important GIB as the first UGIB episode during ICU admission; (2) variceal bleeding related to liver cirrhosis or portal hypertension; (3) pregnancy; (4) palliative care goals at the time of ICU admission; (5) confirmed lower gastrointestinal bleeding or an unclear bleeding source (i.e., unable to be definitively localized to the upper gastrointestinal tract); (6) upper gastrointestinal surgery performed prior to the index UGIB episode or ICU admission for upper gastrointestinal surgery; or (7) prior use of hemostatic agents other than TXA before the index UGIB episode or use of such agents within the 24-hour exposure window after the index UGIB episode, including but not limited to carbazochrome sodium sulfonate, batroxobin, and topical thrombin. For patients who experienced more than one episode of UGIB during the same ICU admission, only the first episode of non-clinically important non-variceal UGIB was included in the analysis.

### 2.3 Definitions of bleeding

UGIB was identified by clinical manifestations including hematemesis, coffee-ground gastric aspirate or emesis, and melena. For patients presenting with melena, available clinical records, imaging findings, endoscopic reports, and physician documentation were reviewed to exclude lower gastrointestinal bleeding whenever possible. Bleeding episodes were classified as variceal or non-variceal based on the same comprehensive review of all available clinical information, including endoscopic reports, imaging studies, documented history of liver disease or portal hypertension, and the treating physician's clinical diagnosis.

Clinically important GIB was defined as overt gastrointestinal bleeding accompanied by at least one of the following: a decrease in hemoglobin level of ≥2 g/dL within 24 h in the absence of another documented source of blood loss; requirement for endoscopic, radiological, or surgical intervention for bleeding control within 24 h after bleeding onset (18).

Non-clinically important non-variceal UGIB was defined as overt UGIB without evidence of variceal bleeding and without meeting the criteria for clinically important bleeding.

### 2.4 Exposure definition and data collection

Time zero was defined as the time of the first documented episode of non-clinically important non-variceal UGIB. The 24-hour landmark was defined as 24 h after time zero. Baseline eligibility criteria were determined using information available at or before the index UGIB episode. In the primary 24-hour landmark analysis, patients who developed clinically important GIB, died, were discharged, or were lost to follow-up before the 24-hour landmark were excluded from both the TXA and non-TXA groups according to the same rule. At the 24-hour landmark, patients were classified as exposed if they had received intravenous TXA within the first 24 h after the index UGIB episode and had not met the criteria for clinically important bleeding before the first dose, and as non-exposed if they had not received TXA or any other systemic hemostatic agent within the same 24-hour window. Exposure status was determined at the 24-hour landmark and was not updated thereafter. The 24-hour landmark exclusion rule and exposure definition were prespecified and applied identically to all patients, regardless of subsequent TXA status. Follow-up for the primary outcome began at the 24-hour landmark in both groups. This landmark approach ensured that exposure status was established before the start of follow-up, prevented immortal time bias, and preserved the temporal sequence of exposure preceding outcome.

The decision to administer TXA, including dose, frequency, and duration, was made by the treating physicians according to clinical judgement. Information on the duration of TXA use, total dose, and mean daily dose was extracted from medication administration records.

Clinical data were extracted from the electronic medical record system. Baseline variables were ascertained using information available at or before the index UGIB episode (time zero), unless otherwise specified. Baseline variables included age, sex, type of ICU admission, admission diagnosis, and major comorbidities. Disease severity was assessed using the Sequential Organ Failure Assessment-2 (SOFA-2) score (19) and the Acute Physiology and Chronic Health Evaluation II (APACHE II) score. The SOFA-2 score was calculated at the 24-hour landmark using the worst values obtained between time zero and the 24-hour landmark. APACHE II scores were recorded within 24 h after ICU admission. Laboratory variables included haemoglobin, platelet count, prothrombin time, activated partial thromboplastin time, international normalized ratio, fibrinogen degradation products, D-dimer, fibrinogen, creatinine, urea, albumin, bilirubin, inflammatory markers, white blood cell count, and lactate. For laboratory variables, values obtained between time zero and the 24-hour landmark were used; if multiple measurements were available, the worst value was selected. Because this was a retrospective study, the exact timing of some laboratory measurements could not always be fully verified; therefore, laboratory values were included based on their recorded collection time in the electronic medical record, and values recorded after the 24-hour landmark were not used. Medication variables were assessed at the 24-hour landmark and included proton pump inhibitors, somatostatin, glucocorticoids, anticoagulants, use of vasoactive-inotropic drugs, mechanical ventilation status, and transfusion therapy administered between time zero and the 24-hour landmark. TXA was considered the exposure of interest and was not included as a baseline medication. Bleeding-related variables included time from ICU admission to the index UGIB episode, clinical manifestation of bleeding, endoscopy after 24-hour landmark, transfusion after 24-hour landmark, and progression to clinically important bleeding. Progression to clinically important bleeding was the primary outcome and was ascertained from the 24-hour landmark onward. Duration of bleeding within 28 days after onset was recorded as follow-up data.

### 2.5 Outcomes

The primary outcome was progression from non-clinically important non-variceal UGIB to clinically important GIB.

Secondary outcomes were assessed from the 24-hour landmark onward. They included duration of UGIB within 28 days after 24-hour landmark, endoscopy requirement, red blood cell transfusion, plasma transfusion, in-hospital mortality, hospital length of stay, duration of mechanical ventilation, 28-day ventilator-free days, and thrombotic complications. Thrombotic complications included deep vein thrombosis, pulmonary embolism, and myocardial infarction. For transfusion outcomes, we recorded the proportion of patients receiving transfusion, transfusion frequency, and transfused volume after the 24-hour landmark.

28 day ventilator-free days were defined as the number of days alive and free from mechanical ventilation during the 28 days after the 24-hour landmark. Patients who died within this 28-day period were assigned a value of 0. Patients who were never ventilated and survived to day 28 after the landmark were assigned a value of 28. Patients who were ventilated but survived to day 28 after the landmark had ventilator-free days calculated as 28 minus the total number of days on mechanical ventilation during this period.

In-hospital mortality was defined as death from any cause during the index hospitalization. Patients who were discharged directly from the ICU were classified as deaths, as such discharges in our institution typically occurred due to treatment withdrawal or clinical futility. Patients who were transferred out of the ICU to the general ward were considered alive and were followed until hospital discharge or death.

### 2.6 Propensity score matching

Because treatment allocation was not randomized, propensity score matching (PSM) was performed to reduce baseline differences between patients who received TXA and those who did not. The propensity score was estimated using a multivariable logistic regression model with TXA use as the dependent variable. Covariates included in the propensity score model were selected based on clinical relevance and their potential influence on the decision to administer TXA. These variables included age, sex, SOFA-2 score, hemoglobin, type of ICU admission (medical vs. surgical), time from ICU admission to the 24-hour landmark, mechanical ventilation status, vasoactive-inotropic drug use, proton pump inhibitor use, anticoagulant use, neurological disease, and severe acute pancreatitis. Patients in the TXA group were matched 1:1 with patients in the non-TXA group using the nearest-neighbour matching method without replacement. A caliper width of 0.2 standard deviations of the logit of the propensity score was applied. Covariate balance before and after matching was assessed using standardized mean differences. An absolute standardized mean difference of <0.10 was considered to indicate adequate balance. The primary and secondary outcomes were compared between the matched groups.

### 2.7 Inverse probability of treatment weighting analysis

Inverse probability of treatment weighting (IPTW) was performed as a sensitivity analysis to assess the robustness of the findings. A propensity score for TXA use was estimated using a multivariable logistic regression model incorporating the same covariates as those used in the PSM model. Stabilized weights were calculated as the inverse probability of receiving the treatment actually administered, extreme propensity scores were trimmed at 0.05 and 0.95, and weights were capped at 10 to reduce the influence of extreme values. Standardized mean differences (SMDs) were used to assess covariate balance between groups after weighting, with an absolute SMD <0.10 indicating acceptable balance. For variables that remained imbalanced (SMD ≥0.10) after IPTW, these covariates were additionally adjusted for in the outcome regression models to further reduce residual confounding (doubly robust estimation). For baseline comparisons after weighting, weighted chi-square tests and weighted independent-sample tests were used for descriptive purposes. For outcome analyses, weighted logistic regression models with robust variance estimation were used for binary outcomes, and weighted Cox proportional hazards models with robust variance estimation were used for time-to-event outcomes, accounting for the weighting structure and providing valid statistical inference.

### 2.8 Regression analysis and subgroup analyses

Exploratory conditional logistic regression analyses were performed in the propensity score-matched cohort to identify factors associated with clinically important bleeding, with stratification by matched pairs to account for the paired structure. Exploratory univariable conditional logistic regression was first used to examine the association between each candidate variable and clinically important bleeding. Variables of clinical importance and variables associated with the outcome in univariate analysis were then considered for multivariable logistic regression. Two Exploratory multivariable conditional logistic regression models were constructed. Model 1 included age, SOFA-2 score, TXA use, and anticoagulant use. Model 2 included shock status assessed at the 24-hour landmark, proton pump inhibitor use, TXA use, and anticoagulant use. Results were reported as odds ratios with 95% confidence intervals.

Predefined subgroup analyses were performed to explore whether the association between TXA use and clinical outcomes differed across clinically relevant patient groups. Subgroups were defined according to SOFA-2 score ≥6 calculated at the 24-hour landmark, presence of shock at the 24-hour landmark, presence of sepsis at the 24-hour landmark, anticoagulant and PPI use during the first 24 h (time zero to 24-hour landmark), neurological disorders, and severe acute pancreatitis as the primary ICU diagnosis. These subgroup analyses were considered exploratory and were performed to generate hypotheses rather than to confirm treatment effects. Within each subgroup, the primary outcome and selected secondary outcomes were compared between the TXA and non-TXA groups. Interaction analyses were performed as the primary criterion for assessing effect modification, with interaction *P* values reported for each subgroup variable. Individual subgroup effects were reported for descriptive purposes only and were not used to draw conclusions regarding differential treatment effects.

### 2.9 Statistical analysis

Continuous variables were presented as mean ± standard deviation or median with interquartile range, according to their distribution. Categorical variables were presented as numbers and percentages. Continuous variables were presented as mean ± standard deviation (SD), and categorical variables as frequencies with percentages, between-group differences were assessed using Student's t-test or Mann–Whitney U test for continuous variables, and chi-square test or Fisher's exact test for categorical variables, as appropriate. For post-matching comparisons, paired analytical methods were used to account for the matched-pair design: paired t-test for continuous variables, and McNemar's test for categorical variables, as appropriate. All statistical tests were two-sided. A *P* value <0.05 was considered statistically significant. Statistical analyses were performed using SPSS Statistics version 26.0 and GraphPad Prism version 9.5.

## 3 Results

### 3.1 Patient selection and baseline characteristics

Between January 2018 and December 2022, a total of 45,088 adult patients were admitted to the intensive care unit (ICU). Among them, 6,022 patients developed UGIB during their ICU stay. After excluding patients with acute UGIB within 48 h after ICU admission, variceal bleeding related to liver cirrhosis, upper gastrointestinal surgery performed prior to the index UGIB episode or ICU admission for upper gastrointestinal surgery, and clinically important UGIB as the initial bleeding episode, 2,096 patients were identified as having non-clinically important non-variceal UGIB. An additional 282 patients were excluded because they received haemostatic agents other than TXA. Finally, 1,814 patients were included in the analysis, of whom 154 received TXA after the onset of bleeding and 1,660 did not receive TXA or other haemostatic agents (Figure 1).

**Figure 1:**
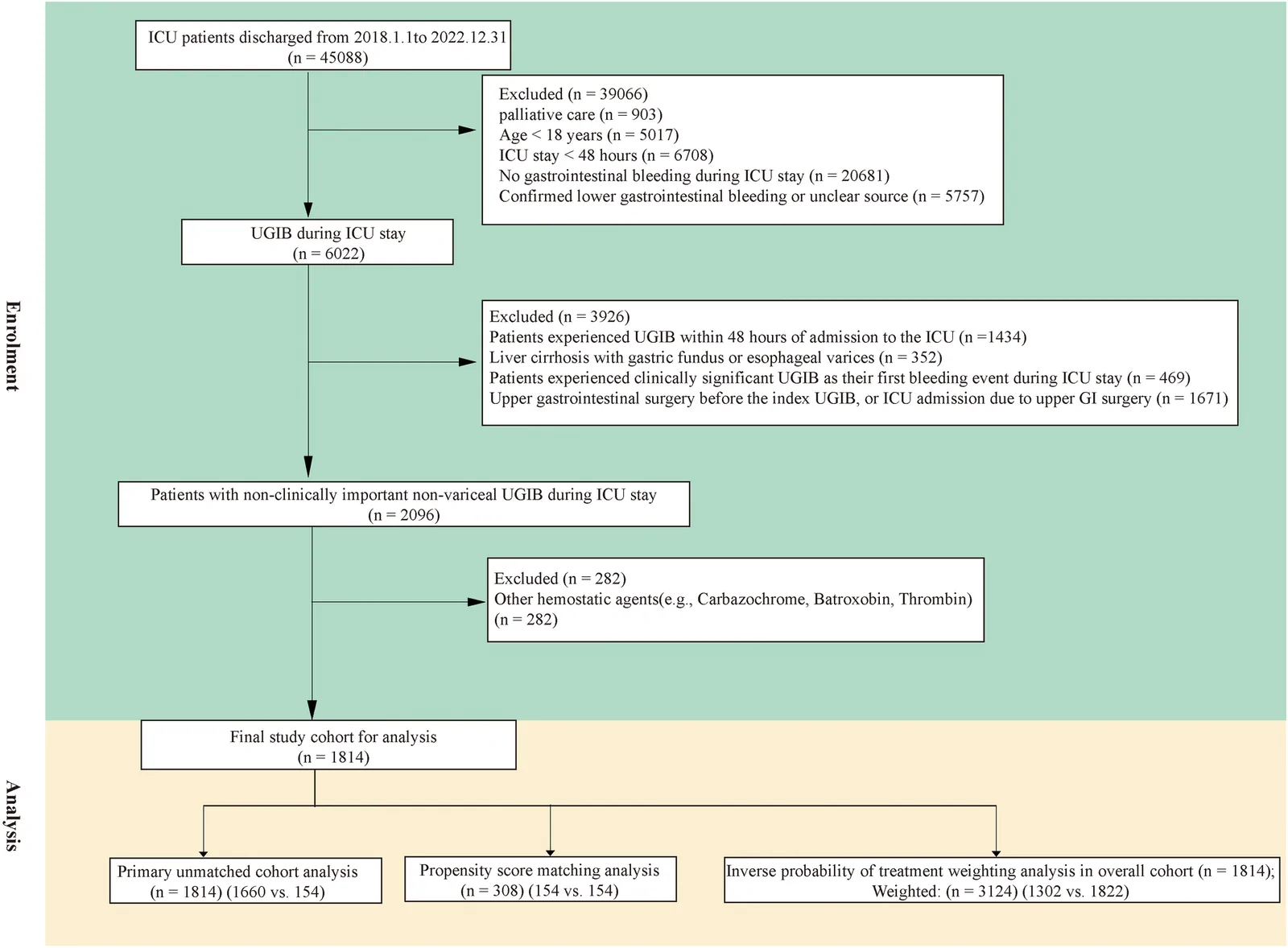
Flowchart of current study. A total of 45,088 adult patients were admitted to the intensive care unit between January 2018 and December 2022. Among them, 6,022 patients developed UGIB during ICU hospitalization. After excluding patients with acute UGIB within 48 h after ICU admission, variceal bleeding related to liver cirrhosis, upper gastrointestinal surgery performed prior to the index UGIB episode or ICU admission for upper gastrointestinal surgery, and clinically important UGIB as the initial bleeding episode, 2,096 patients with non-clinically important non-variceal UGIB were identified. After further excluding 282 patients who received hemostatic agents other than TXA, 1,814 patients were included in the final analysis. Of these, 154 patients received TXA after bleeding onset, and 1,660 patients did not receive TXA or other hemostatic agents.

### 3.2 Baseline characteristics before and after propensity score matching

Before PSM, several baseline differences were observed between the TXA and Non-TXA groups. Patients in the TXA group were more frequently admitted for medical conditions than those in the Non-TXA group. The proportion of patients with neurological disorders was lower in the TXA group, whereas severe acute pancreatitis was more common in the TXA group (Table 1). The TXA group had a longer time from ICU admission to the 24-hour landmark and a lower worst haemoglobin value between time zero and the 24-hour landmark. PPI use at the 24-hour landmark was also less frequent in the TXA group. (Table 2).

**Table 1:** Baseline characteristics before and after PSM.

Parameters (mean ± SD) / median (IQR)	Entire cohort				PSM cohort			
	TXA group (*n* = 154)	Non-TXA group (*n* = 1,660)	*P*-value	SMD	TXA group (*n* = 154)	Non-TXA group (*n* = 154)	*P*-value	SMD
Age, y	55.01 ± 15.81	54.38 ± 16.86	0.641	0.038	55.01 ± 15.81	53.64 ± 17.50	0.460	0.082
Male n%	113 (73.4%)	1,150 (69.3%)	0.290	0.091	113 (73.4%)	114 (74.0%)	0.897	−0.015
APACHE Ⅱ	20.96 ± 6.78	18.50 ± 8.66	<0.001	0.317	20.96 ± 6.78	21.05 ± 8.74	0.925	−0.011
Type of admission			＜0.001	0.337			0.683	0.062
Medical n%	94 (61.0%)	737 (44.4%)			94 (61.0%)	90 (58.4%)		
Surgical n%	60 (39.0%)	923 (55.6%)			60 (39.0%)	64 (41.6%)		
Complication								
Diabetes n%	16 (10.4%)	181 (10.9%)	0.952	−0.017	16 (10.4%)	17 (11.0%)	>0.999	0.021
CKD n%	5 (3.2%)	76 (4.6%)	0.575	−0.069	5 (3.2%)	6 (3.9%)	>0.999	−0.035
Acute coronary syndrome n%	9 (5.8%)	157 (9.5%)	0.187	−0.136	9 (5.8%)	13 (8.4%)	0.503	−0.084
Severe pneumonia n%	75 (48.7%)	890 (53.6%)	0.242	−0.098	75 (48.7%)	82 (53.2%)	0.488	0.091
Burn victim n%	0 (0.0%)	7 (0.4%)	>0.999	−0.092	0 (0.0%)	0 (0.0%)	–	–
Cancer n%	23 (14.9%)	182 (11.0%)	0.175	0.118	20 (13.0%)	17 (11.0%)	0.719	0.060
Neurological disorders n%	29 (18.8%)	502 (30.2%)	0.004	−0.267	29 (18.8%)	32 (20.8%)	0.755	−0.057
Severe acute pancreatitis n%	35 (22.7%)	189 (11.4%)	＜0.001	0.304	35 (22.7%)	29 (18.8%)	0.361	0.096
Trauma n%	19 (12.3%)	227 (13.7%)	0.733	−0.041	19 (12.3%)	19 (12.3%)	>0.999	0

CKD, Chronic Kidney Disease.

**Table 2:** Characteristics of the TXA and non-TXA groups at the 24-hour landmark after non-clinically important NVUGIB.

Parameters (mean ± SD)/median (IQR)	Entire cohort				PSM cohort			
	TXA group (*n* = 154)	Non-TXA group (*n* = 1660)	*P*-value	SMD	TXA group (*n* = 154)	Non-TXA group (*n* = 154)	*P*-value	SMD
Severity and clinical status								
Time to Bleeding, d	9 (5, 14)	7 (4, 13)	0.063	0.159	9 (5, 14)	9 (6, 13)	0.844	0.021
SOFA-2, score	5.86 ± 3.83	5.80 ± 3.72	0.838	0.017	5.86 ± 3.83	5.94 ± 4.20	0.880	−0.018
Sepsis, n%	88 (57.1%)	968 (58.3%)	0.761	−0.011	88 (57.1%)	89 (57.8%)	>0.999	−0.002
Shock, n%	71 (46.1%)	788 (47.5%)	0.689	−0.064	71 (46.1%)	72 (46.8%)	>0.999	−0.004
Laboratory findings								
HGB, g/L	89.97 ± 17.59	93.53 ± 20.08	0.019	−0188	89.97 ± 17.59	89.49 ± 18.52	0.806	0.027
PLT, 10^9^/L	190.84 ± 140.33	189.61 ± 134.02	0.917	0.089	190.84 ± 140.33	192.51 ± 141.90	0.917	−0.012
INR	1.27 ± 0.53	1.26 ± 0.41	0.747	0.030	1.27 ± 0.53	1.25 ± 0.30	0.656	−0.049
APTT, sec	35.93 ± 8.26	34.77 ± 10.37	0.103	0.124	35.93 ± 8.26	35.98 ± 16.39	0.973	−0.004
PT, sec	14.02 ± 3.39	13.95 ± 5.09	0.816	0.016	14.02 ± 3.39	14.14 ± 3.18	0.737	−0.035
CREA, umol/L	104.32 ± 102.87	94.12 ± 90.54	0.285	0.095	104.32 ± 102.87	108.35 ± 117.52	0.663	−0.046
UREA, umol/L	12.80 ± 8.18	10.92 ± 9.64	0.008	0.211	12.80 ± 8.18	12.17 ± 12.04	0.618	0.062
ALB, g/L	33.64 ± 5.98	33.59 ± 6.04	0.929	0.007	33.64 ± 5.98	33.42 ± 5.11	0.739	0.001
TBIL, umol/L	40.19 ± 56.40	31.50 ± 50.40	0.066	0.163	40.19 ± 56.40	34.31 ± 60.29	0.381	0.099
DBIL, umol/L	29.06 ± 46.72	20.84 ± 38.97	0.036	0.191	29.06 ± 46.72	24.41 ± 51.46	0.408	0.095
CRP, mg/L	107.32 ± 99.78	104.14 ± 92.54	0.704	0.045	107.32 ± 99.78	104.72 ± 82.82	0.804	0.028
PCT, ng/mL	4.02 ± 7.33	3.18 ± 9.84	0.185	0.098	4.02 ± 7.33	4.36 ± 11.63	0.769	−0.035
WBC, 10^9^/L	11.28 ± 5.48	11.51 ± 6.76	0.617	0.034	11.61 ± 5.28	12.01 ± 7.49	0.596	−0.062
LAC, mmol/L	1.89 ± 1.42	1.92 ± 1.73	0.776	−0.022	1.89 ± 1.42	1.90 ± 1.33	0.932	−0.010
Medications and supportive treatments								
Vasoactive-inotropic drugs n%	41 (26.6%)	530 (31.9%)	0.175	−0.117	41 (26.6%)	39 (25.3%)	0.892	0.030
Max norepinephrine infusion rate, μg/kg/min	0 (0, 0.78)	0 (0, 0.99)	0.965	−0.003	0 (0, 0.78)	0 (0, 0.63)	0.766	−0.034
Mechanical ventilation n%	89 (57.8%)	931 (56.1%)	0.683	0.034	89 (57.8%)	93 (60.4%)	0.743	−0.053
PPI n%	58 (37.7%)	874 (52.7%)	<0.001	−0.304	58 (37.7%)	51 (33.1%)	0.427	0.095
Somatostatin n%	42 (27.3%)	347 (20.9%)	0.065	0.149	42 (27.3%)	40 (26.0%)	0.892	0.029
Anticoagulant drugs n%	21 (13.6%)	241 (14.5%)	0.859	−0.023	21 (13.6%)	18 (11.7%)	0.728	0.058
Enteral Nutrition n%	34 (22.1%)	412 (24.8%)	0.511	−0.064	34 (22.1%)	32 (20.8%)	0.892	0.031
Transfusion on the day of 24-hour landmark								
Red blood cell suspension transfusion n%	3 (1.9%)	13 (0.8%)	0.149	0.113	3 (1.9%)	2 (1.3%)	>0.999	0.051
Plasma transfusion n%	15 (9.7%)	130 (7.8%)	0.496	0.067	15 (9.7%)	11 (7.1%)	0.541	0.093

Time to Bleeding: The number of days from admission to the ICU to the day of 24-hour landmark after non-clinically important NVUGIB.

SOFA 2.0, Sequential Organ Failure Assessment 2.0; HGB, Hemoglobin; RBC, Red Blood Cell; PLT, Platelet; INR, International Normalized Ratio; PT, Prothrombin Time; APTT, Activated Partial Thromboplastin Time; CREA, Creatinine; UREA, Urea; ALB, Albumin; TBIL, Total Bilirubin; DBIL, Direct Bilirubin; IBIL, Indirect Bilirubin; AST, Aspartate Aminotransferase; ALT, Alanine Aminotransferase; CRP, C-Reactive Protein; PCT, Procalcitonin; WBC, White Blood Cell; LAC, Lactate; PPI, Proton Pump Inhibitors.

After PSM, 154 patients in the TXA group were matched with 154 patients in the Non-TXA group. The matched groups were generally comparable in terms of type of ICU admission, SOFA-2 score, proton pump inhibitor use, anticoagulant use, HGB, mechanical ventilation and vasopressor use at the 24-hour landmark time, coexisting neurological diseases, and severe pancreatitis. during the first 24 h (time zero to the 24-hour landmark), no statistically significant differences were observed between the two matched groups in SOFA-2 score, haemoglobin level, platelet count, coagulation parameters, renal function, inflammatory markers, vasoactive-inotropic drug use, mechanical ventilation, proton pump inhibitor use, somatostatin use, anticoagulant use, or transfusion status (Table 2).

### 3.3 Tranexamic acid exposure

Among patients in the TXA group, TXA was administered for a mean duration of 1.71 ± 1.42 days. The mean total dose of TXA was 2.12 ± 2.36 g, and the mean daily dose was 1.21 ± 0.47 g/day (Table 3).

**Table 3:** TXA exposure characteristics.

Parameters (mean ± SD)	TXA group (*n* = 154)
Using TXA days, d	1.71 ± 1.42
Total tranexamic acid dose, g	2.12 ± 2.36
Daily dose, g/d	1.21 ± 0.47

TXA, Tranexamic acid.

### 3.4 Primary outcome

Before PSM, progression to clinically important GIB occurred in 9 patients in the TXA group and 95 patients in the Non-TXA group. The incidence of clinically important bleeding was not significantly different between the two groups (9/154, 5.8% vs. 95/1,660, 5.7%; *P* = 0.951, 95% CI: 0.450–1.707) (Table 4). After PSM, the incidence of clinically important gastrointestinal bleeding remained comparable between the TXA and Non-TXA groups (9/154, 5.8% vs. 12/154, 7.8%; *P* = 0.664, 95% CI: 0.562–3.164). These findings indicate that TXA use was not associated with a reduced risk of progression from non-clinically important non-variceal UGIB to clinically important GIB in critically ill patients.

**Table 4:** Clinical outcomes before and after adjustment.

Parameters (mean ± SD)/median (IQR)	Primary unmatched cohort analysis				PSM cohort analysis				IPTW sensitivity analysis			
	TXA group (*n* = 154)	Non-TXA group (*n* = 1,660)	*P*-value	95% CI	TXA group (*n* = 154)	Non-TXA group (*n* = 154)	*P*-value	95% CI	TXA group (Weighted *n* ≈ 1,302)	Non-TXA group (Weighted *n* ≈ 1,822)	*P*-value	95% CI
Clinically important GIB n%	9 (5.8%)	95 (5.7%)	0.951	0.450–1.707	9 (5.8%)	12 (7.8%)	0.664	0.562–3.164	67 (5.1%)	106 (5.8%)	0.418	0.840–1.522
Days of UGIB within 28 days after 24-hour landmark, d	3 (2, 6)	4 (2, 8)	＜0.001	−1.311–0.139	3 (2, 6)	3 (1, 4)	0.951	0.884–2.636	3 (2, 4)	4 (2, 7)	＜0.001	−1.822–1.294
Endoscopy n%	40 (26.0%)	407 (24.5%)	0.688	0.794–1.384	40 (26.0%)	33 (21.4%)	0.442	0.539–1.388	333 (25.6%)	448 (24.6%)	0.530	0.850–1.087
In-hospital mortality n%	79 (51.3%)	808 (48.7%)	0.533	0.761–1.053	79 (51.3%)	71 (46.1%)	0.734	0.755–1.435	656 (50.4%)	890 (48.8%)	0.397	0.903–1.041
LOS, d	30 (20, 44)	27 (17, 45)	0.645	−3.341–5.425	30 (20, 44)	26 (15, 45)	0.329	−3.100–9.204	30 (19, 45)	28 (18, 44)	0.151	−3.100–9.204
Mechanical Ventilation free in 28 days, d	11 (5, 20)	17 (7, 24)	＜0.001	−4.973–−1.776	11 (5, 20)	18 (8, 25)	0.019	−6.399–−1.893	12 (1, 20)	17 (7, 24)	＜0.001	−4.131–−2.768
Mechanical Ventilation time, d	9 (5, 14)	7 (4, 13)	0.006	1.178–7.236	9 (5, 14)	9 (3, 20)	0.266	0.923–8.976	16 (8, 27)	11 (4, 21)	＜0.001	3.216–5.842
RBC transfused post-bleeding n%	11 (7.1%)	51 (3.1%)	0.017	0.210–0.710	12 (7.8%)	11 (7.1%)	0.832	0.360–1.929	81 (6.2%)	104 (5.7%)	0.591	0.391–1.724
Among transfused patients: volume, U	3 (2, 4.5)	2 (2, 3)	0.771	−0.945–1.251	3 (2, 4.5)	3 (2, 4.5)	>0.999	−2.410–1.301	3 (2, 4.5)	2.5 (1.5, 4)	0.578	−2.410–1.301
Among transfused patients: frequency, times	2 (1, 3)	1 (1, 2)	0.891	−0.654–1.747	2 (1, 3)	2 (1, 3.5)	0.943	−1.926–0.945	2 (1, 3)	2 (1, 2)	0.846	−1.926–0.945
Plasma transfused post-bleeding n%	21 (13.6%)	89 (5.4%)	0.003	0.510–0.907	21 (13.6%)	21 (13.6%)	0.732	0.357–1.731	192 (14.8%)	287 (15.8%)	0.450	0.596–1.934
Among transfused patients: volume, mL	400 (350, 850)	450 (250, 825)	0.818	−252.265–200.472	400 (350, 850)	550 (400, 1150)	0.127	−416.244–354.339	400 (350, 800)	500 (400, 950)	0.180	−284.365–161.460
Among transfused patients: frequency, times	1 (1, 2)	2 (1, 4)	0.014	−2.090–−0.571	1 (1, 2)	3 (2, 3)	0.313	−4.582–0.867	1 (1, 2)	2 (1, 4)	＜0.001	−2.019–−0.553
Deep vein thrombosis n%	18 (11.7%)	300 (18.1%)	0.060	0.996–2.431	18 (11.7%)	26 (16.9%)	0.256	0.758–2.628	126 (9.7%)	326 (17.9%)	＜0.001	1.525–2.243
Pulmonary embolism n%	3 (1.9%)	19 (1.1%)	0.384	0.196–2.153	3 (1.9%)	1 (0.6%)	0.625	0.035–3.205	22 (1.7%)	20 (1.1%)	0.210	0.357–1.187
Myocardial infarction n%	9 (5.8%)	32 (1.9%)	0.004	0.150–0.639	9 (5.8%)	5 (3.2%)	0.344	0.204–1.910	73 (5.6%)	35 (1.9%)	0.001	0.231–0.510

LOS, length of stay; GIB, Gastrointestinal Bleeding; RBC, Red blood cell.

### 3.5 Secondary outcomes in the matched cohort

In the propensity score-matched cohort, there were no significant differences between the TXA and Non-TXA groups in the duration of UGIB within 28 days (3 (2, 6) vs. 3 (1, 4) days; *P* = 0.951, 95% CI: 0.884–2.636), endoscopy requirement (26.0% vs. 21.4%; *P* = 0.442, 95% CI: 0.539–1.388), red blood cell transfusion after 24-hour landmark, plasma transfusion after 24-hour landmark, in-hospital mortality (51.3% vs. 46.1%; *P* = 0.734, 95% CI: 0.562–3.164), or hospital length of stay (30 (20, 44) vs. 26 (15, 45) days; *P* = 0.329, 95% CI: −3.100–9.204) (Table 4). The duration of mechanical ventilation was numerically longer in the TXA group than in the Non-TXA group, although the difference did not reach statistical significance (9 (5, 14) vs. 9 (3, 20) days; *P* = 0.266, 95% CI: 0.923–8.976). However, 28-day ventilator-free days were significantly lower in the TXA group (11 (5, 20) vs. 18 (8, 25) days; *P* = 0.019, 95% CI: −6.399–−1.893) (Table 4).

Thrombotic events were not significantly different between the two groups after matching. Deep vein thrombosis occurred in 18 patients in the TXA group and 26 patients in the Non-TXA group (11.7% vs. 16.9%; *P* = 0.256, 95% CI: 0.758–2.628). Pulmonary embolism occurred in 3 patients and 1 patient, respectively (1.9% vs. 0.6%; *P* = 0.625, 95% CI: 0.035–3.205), and myocardial infarction occurred in 9 patients and 5 patients, respectively (5.8% vs. 3.2%; *P* = 0.344, 95% CI: 0.204–1.910) (Table 4). Survival analysis showed no significant difference in hospital survival between the TXA and Non-TXA groups after PSM, with a log-rank *P* value of 0.610 (Figure 3).

### 3.6 Changes in hemoglobin and coagulation-related parameters

The dynamic changes in hemoglobin and coagulation-related parameters before and after the 24-hour landmark are shown in Figure 2. Hemoglobin, INR, fibrinogen degradation products, and D-dimer were compared between the TXA and Non-TXA groups from 3 days before to 12 days after the bleeding event, The interval from time zero to the 24-hour landmark was defined as Day 0 (0–24 h after the index UGIB episode) (Figures 2A–D). Additional changes in coagulation-related parameters, inflammatory markers, and albumin are presented in the Supplementary Figures.

**Figure 2:**
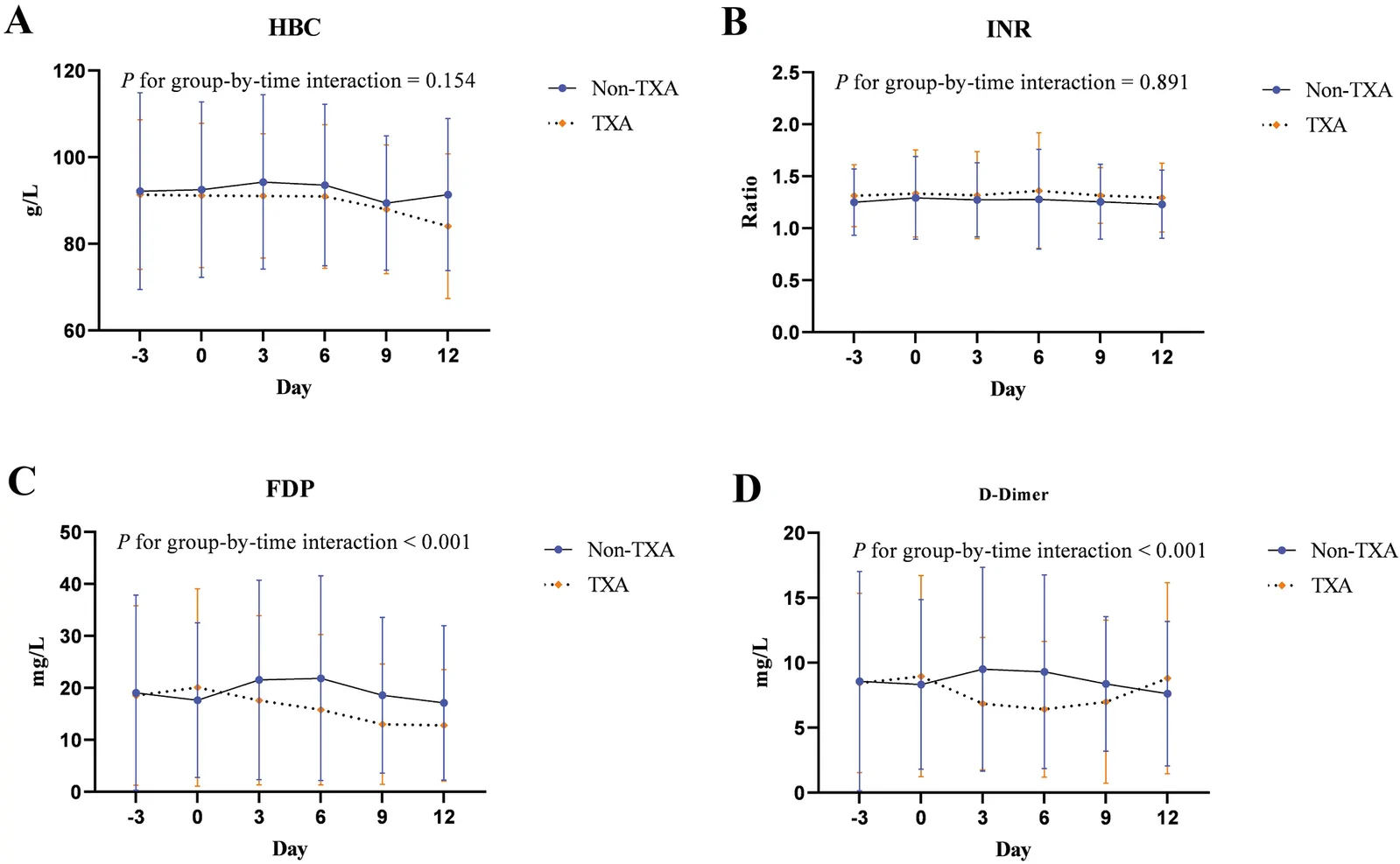
Dynamic changes in hemoglobin and coagulation-related parameters before and after non-clinically important non-variceal UGIB. The figure shows the dynamic changes in hemoglobin and coagulation-related parameters from 3 days before to 12 days after the onset of non-clinically important non-variceal UGIB. Day 0: 0–24 h after the index UGIB episode (time zero to the 24-hour landmark). **(A)** Changes in hemoglobin levels. **(B)** Changes in international normalized ratio. **(C)** Changes in fibrinogen degradation products. **(D)** Changes in D-dimer levels. UGIB, upper gastrointestinal bleeding; HGB, haemoglobin; INR, international normalized ratio; FDP, fibrinogen degradation products; TXA, tranexamic acid. * *p* < 0.05, ** *p* < 0.01, *** *p* < 0.001, ns: non-significant.

**Figure 3:**
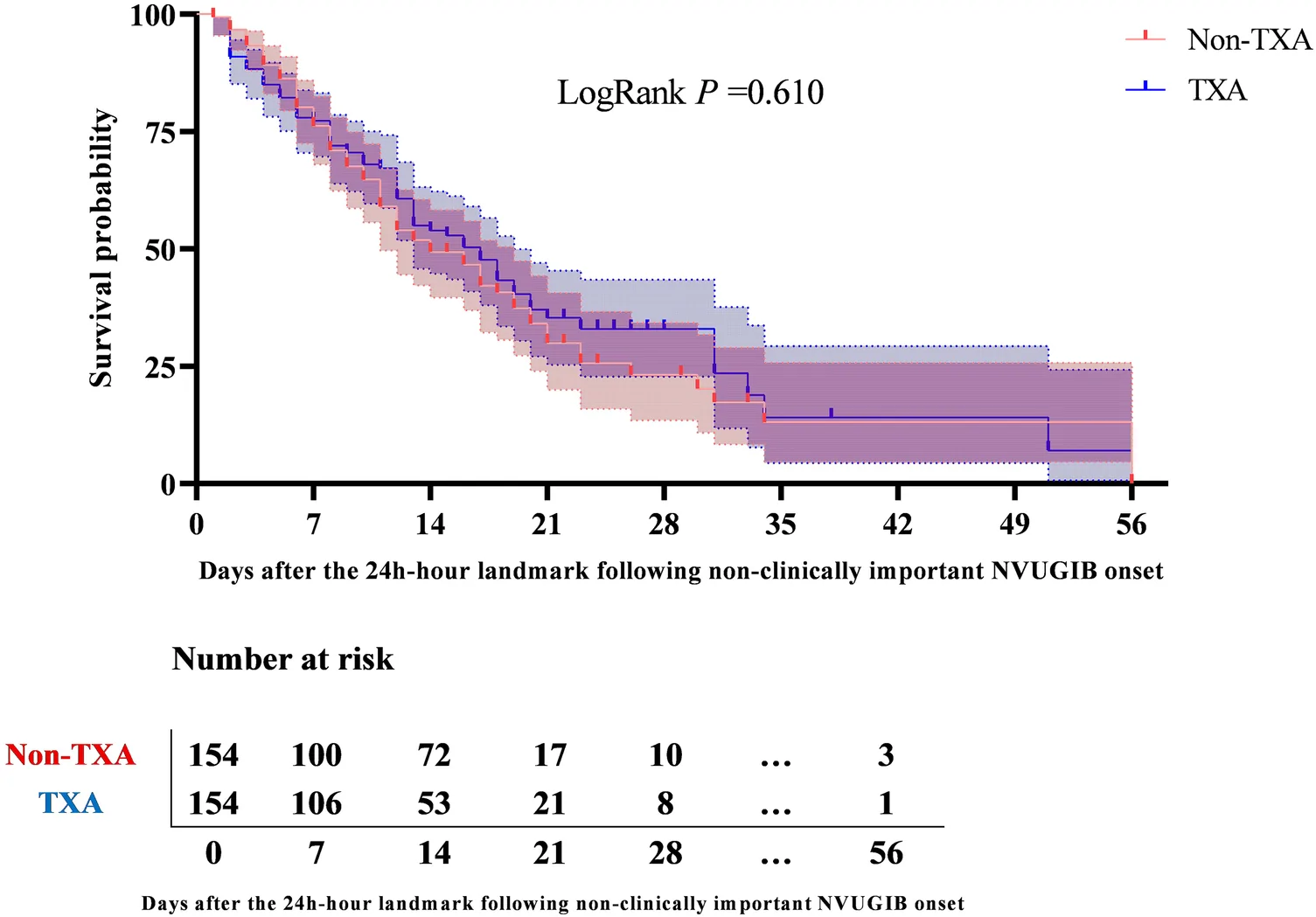
Survival curves of the TXA and Non-TXA groups after propensity score matching. Kaplan–Meier survival curves comparing in-hospital survival between patients who received TXA and those who did not receive TXA after propensity score matching. No significant difference in survival was observed between the TXA and Non-TXA groups.

### 3.7 Inverse probability of treatment weighting analysis

Inverse probability of treatment weighting (IPTW) was performed as a sensitivity analysis in the original cohort (*n* = 1,814). Baseline characteristics are presented in Supplementary Tables 1 and 2. After weighting, the TXA group (n ≈ 1,302) and non-TXA group (n ≈ 1,822) were analyzed, the incidence of clinically important gastrointestinal bleeding remained similar between the TXA and Non-TXA groups (5.1% vs. 5.8%; *P* = 0.418, 95% CI: 0.840–1.522), consistent with the propensity score-matched analysis (Table 4).

There were also no significant differences in endoscopy requirement (25.6% vs. 24.6%; *P* = 0.530, 95% CI: 0.850–1.087), in-hospital mortality (50.3% vs. 48.8%; *P* = 0.397, 95% CI: 0.903–1.041), or hospital length of stay (36.58 ± 26.73 vs. 35.10 ± 30.89 days; *P* = 0.151, 95% CI: −3.100–9.204) between the weighted groups (Table 4). However, several secondary outcomes showed findings that diverged from the PSM results, The TXA group also had fewer 28-day ventilator-free days (12 (1, 20) vs. 17 (7, 24) days; *P* < 0.001, 95% CI: −4.131–−2.768) and longer mechanical ventilation duration (16 (8, 27) vs. 11 (4, 21) days; *P* < 0.001, 95% CI: 3.216–5.842), deep vein thrombosis (9.7% vs. 17.9%; *P* < 0.001, 95% CI: 1.525–2.243), and myocardial infarction (5.6% vs. 1.9%; *P* = 0.001, 95% CI: 0.231–0.510), all showed statistically significant differences favoring the Non-TXA group, whereas the PSM analysis did not detect significant differences for these outcomes (Table 4).

### 3.8 Risk factors for progression to clinically important gastrointestinal bleeding

Exploratory univariate logistic regression analysis in the matched cohort showed that a higher SOFA-2 score was associated with progression to clinically important gastrointestinal bleeding. TXA use was not associated with progression to clinically important bleeding in univariate analysis (OR 1.333, 95% CI 0.562–3.164; *P* = 0.514) (Table 5). Two exploratory multivariable conditional logistic regression models were then constructed. In Model 1, after adjustment for age, SOFA-2 score, TXA use, and anticoagulant use, TXA was not significantly associated with clinically important gastrointestinal bleeding (OR 1.067, 95% CI 0.394–2.889; *P* = 0.899) (Figure 4A), SOFA-2 score showed a statistically significant association with clinically important gastrointestinal bleeding (OR 1.169, 95% CI 1.002–1.369; *P* = 0.042). In Model 2, after adjustment for shock, proton pump inhibitor use, TXA use, and anticoagulant use, TXA use remained non-significant (OR 1.505, 95% CI 0.595–3.809; *P* = 0.388) (Figure 4B). Given the limited number of primary outcome events, these multivariable models should be considered exploratory, and the results should be interpreted as hypothesis-generating.

**Table 5:** Exploratory univariate conditional logistic regression analysis of factors associated with progression to clinically important GIB.

Variable	OR	95%CI	*P*-value
Sex	1.000	0.202–4.955	>0.999
Age	1.009	0.976–1.044	0.602
Type of admission (Ref Medical)	1.000	0.250–3.998	>0.999
SOFA-2 score	1.161	1.001–1.347	0.048
Using anticoagulation drugs	1.000	0.063–15.988	>0.999
Somatostatin	0.500	0.092–2.730	0.423
Plasma transfusion	0.333	0.035–3.205	0.341
shock	0.444	0.137–1.443	0.177
Using PPI after 24-hour landmark	0.600	0.143–2.511	0.484
Enteral Nutrition	2.000	0.366–10.919	0.423
TXA	1.333	0.562–3.164	0.514
APACHE Ⅱ	1.045	0.975–1.119	0.216
Sepsis	0.222	0.048–1.028	0.054
Neurological disorders	2.000	0.368–10.921	0.423
Severe acute pancreatitis	0.667	0.111–3.990	0.657
Severe pneumonia	0.500	0.151–1.660	0.258
cancer	4.000	0.447–35.788	0.215

SOFA-2, Sequential Organ Failure Assessmen-2; LOS, length of stay; APACHE II, Acute Physiology and Chronic Health Evaluation II; PPI, Proton Pump Inhibitors.

**Figure 4:**
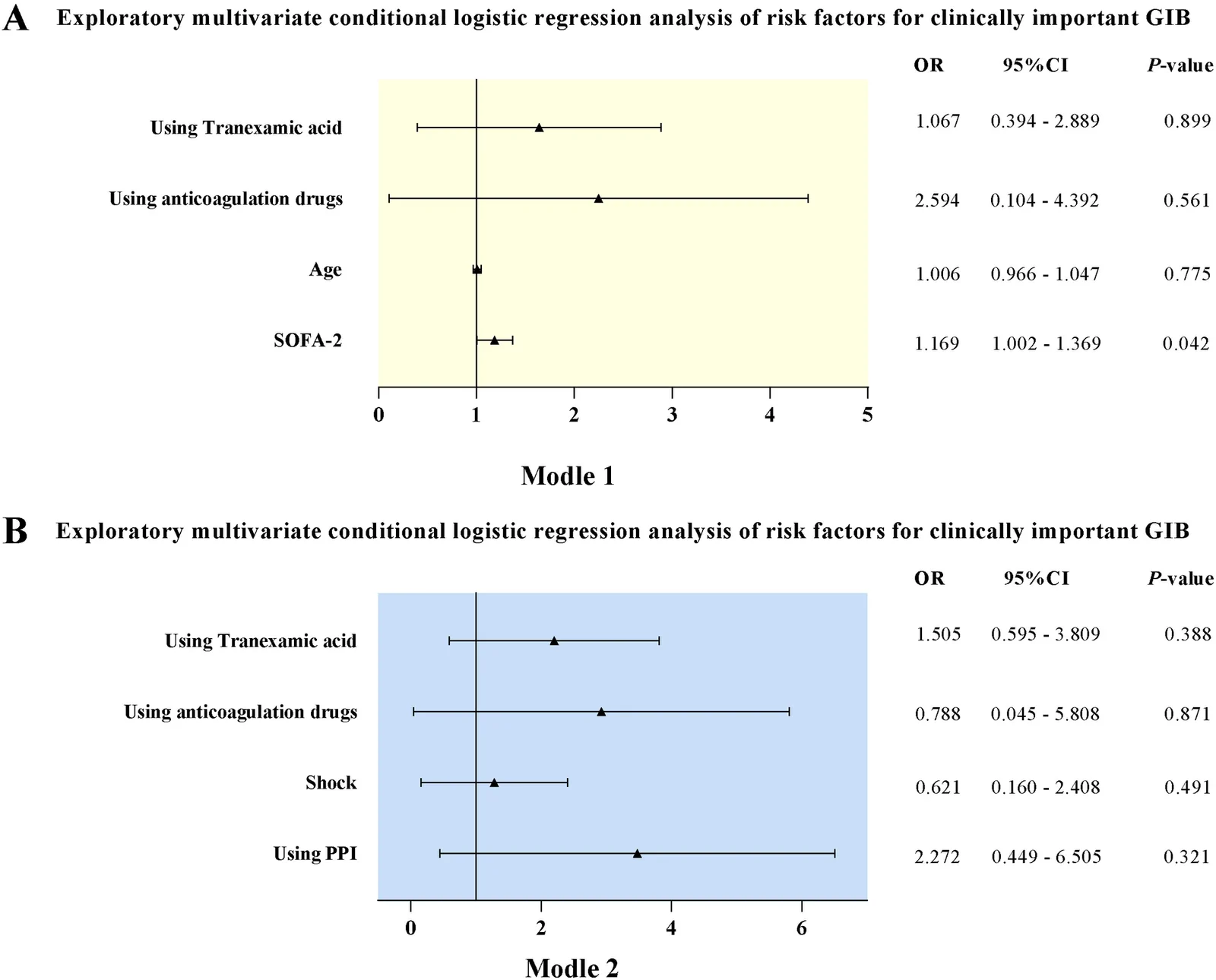
Exploratory multivariable logistic regression analysis of factors associated with clinically important gastrointestinal bleeding. Forest plots showing the multivariable logistic regression analysis of factors associated with progression to clinically important gastrointestinal bleeding in critically ill patients with non-clinically important non-variceal UGIB. **(A)** Model 1 was adjusted for age, SOFA-2 score, TXA use, and anticoagulant use assessed at the 24-hour landmark. **(B)** Model 2 was adjusted for shock, proton pump inhibitor use, and anticoagulant use assessed at the 24-hour landmark. GIB, gastrointestinal bleeding; SOFA, Sequential Organ Failure Assessment; TXA, tranexamic acid; PPI, proton pump inhibitor; OR, odds ratio; CI, confidence interval.

### 3.9 Subgroup analyses

Subgroup analyses were performed according to SOFA-2 score ≥ 6, shock, sepsis, anticoagulant and PPI use, and neurological disorders and severe acute pancreatitis as the primary ICU diagnoses at admission. Across all predefined subgroups, TXA use was not associated with a significant reduction in the incidence of clinically important gastrointestinal bleeding (Tables 6; Figure 5).

**Table 6:** Subgroup analysis for clinically important GIB.

Subgroup	TXA group	Non-TXA group	*P*-value
SOFA −2 ≥ 6	(*n* = 73)	(*n* = 64)	
	5 (6.8%)	8 (12.5%)	0.404
Shock status	(*n* = 71)	(*n* = 72)	
	6 (8.5%)	6 (8.3%)	>0.999
Sepsis	(*n* = 88)	(*n* = 89)	
	7 (8.0%)	8 (9.0%)	>0.999
Using anticoagulants	(*n* = 21)	(*n* = 18)	
	1 (4.8%)	0	>0.999
Neurological disorders	(*n* = 29)	(*n* = 36)	
	1 (3.4%)	2 (5.6%)	>0.999
Severe acute pancreatitis	(*n* = 35)	(*n* = 29)	
	3 (8.6%)	4 (13.8%)	0.692
Using PPI	(*n* = 58)	(*n* = 51)	
	5 (8.6%)	6 (11.8%)	0.822

SOFA-2, Sequential Organ Failure Assessment-2; PPI, Proton Pump Inhibitors.

**Figure 5:**
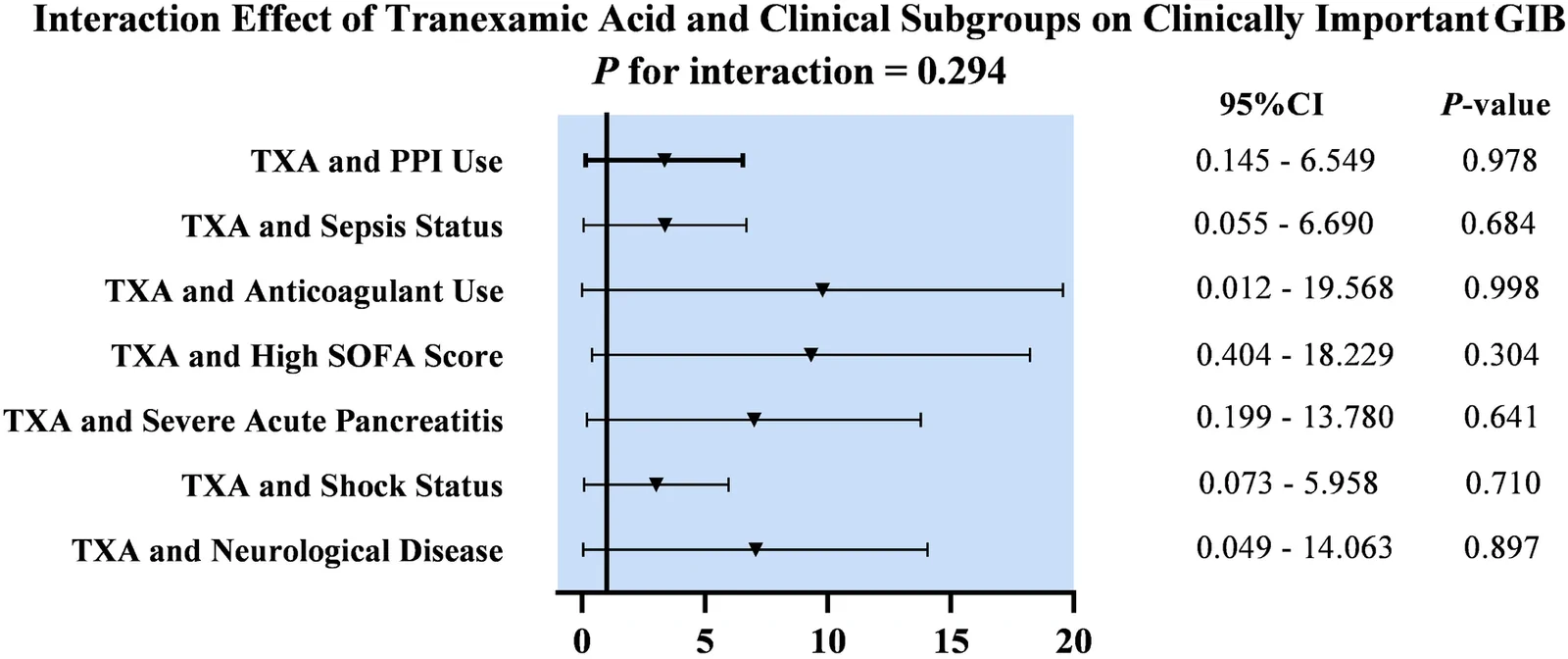
Subgroup analysis of the association between TXA use and clinically important gastrointestinal bleeding. Forest plot shows the association between TXA use and progression to clinically important gastrointestinal bleeding across predefined clinical subgroups. Subgroups were defined according to SOFA-2 score ≥6 calculated at the 24-hour landmark; shock, sepsis, and anticoagulant use assessed at the 24-hour landmark; neurological disorders as the primary ICU diagnosis; and severe acute pancreatitis as the primary ICU diagnosis. No significant interaction was observed between TXA use and the predefined subgroups for clinically important gastrointestinal bleeding. TXA, tranexamic acid; GIB, gastrointestinal bleeding; SOFA, Sequential Organ Failure Assessment; ICU, intensive care unit; OR, odds ratio; CI, confidence interval.

In patients with SOFA-2 score ≥ 6, the incidence of clinically important gastrointestinal bleeding was 6.8% in the TXA group and 12.5% in the Non-TXA group (*P* = 0.404). In patients with shock, the corresponding rates were 8.5% and 8.3% (*P* > 0.999). In patients with sepsis, clinically important bleeding occurred in 8.0% and 9.0% of patients, respectively (*P* > 0.999). No significant differences in clinically important bleeding were observed among patients receiving anticoagulants or PPI, patients with neurological disorders, or patients with severe acute pancreatitis (Tables 6).

Most secondary outcomes were comparable between the TXA and non-TXA groups across all predefined subgroups. No significant interactions were observed between TXA use and any subgroup variable for clinically important bleeding (Figures 5; *P* for interaction = 0.294). Although a few comparisons showed nominal differences in 28-day ventilator-free days (e.g., in patients with SOFA-2 score ≥6 or PPI use; Supplementary Tables 17 and 23), these isolated findings should be interpreted with extreme caution given the exploratory nature of these analyses, the limited number of primary outcome events, and the potential for type I error due to multiple comparisons. No consistent pattern of differential treatment effects was observed across subgroups.

## 4 Discussion

In this retrospective cohort study of critically ill patients with non-clinically important non-variceal UGIB, TXA use was not associated with a statistically detectable reduction in the risk of progression to clinically important GIB. This finding was consistent in both the propensity score-matched cohort. In the matched cohort, TXA use was also not associated with significant reductions in endoscopy requirement, transfusion requirement, duration of bleeding, hospital length of stay, in-hospital mortality, or thrombotic complications. However, patients receiving TXA had fewer 28-day ventilator-free days in the matched cohort, but this finding should be interpreted cautiously given the exploratory nature of these analyses and the limited number of events. Taken together, these findings do not support a clinically meaningful benefit of routine empirical TXA use for non-clinically important non-variceal UGIB in critically ill patients, although a modest benefit or harm cannot be excluded given the width of the confidence intervals.

TXA has become an important component of blood conservation strategies in several clinical settings, particularly in perioperative medicine. Its antifibrinolytic mechanism provides a strong biological rationale for preventing clot breakdown and reducing bleeding (6). In surgical patients, bleeding is usually anticipated, temporally well defined, and related to a controlled procedural injury. Under these conditions, TXA can be administered before or during the period of maximal fibrinolytic activation, which may partly explain its favourable effects in reducing perioperative blood loss and transfusion requirements (9, 10, 12). In contrast, UGIB in critically ill patients is substantially more heterogeneous. The onset of bleeding is often uncertain, the bleeding source may be multifactorial, and the timing of recognition may be delayed. Therefore, although the success of TXA in perioperative bleeding supports its haemostatic potential, these benefits cannot be automatically extrapolated to ICU patients with non-clinically important non-variceal UGIB.

Several mechanisms may explain the lack of apparent benefit observed in the present study. First, non-clinically important non-variceal UGIB in the ICU is often mild and self-limited (20). Many episodes manifest as coffee-ground gastric aspirate, mild haematemesis, or melena without haemodynamic instability or substantial haemoglobin decline. In such cases, spontaneous haemostasis may already have occurred by the time bleeding is recognized, limiting the opportunity for TXA to exert a meaningful effect. Second, the pathophysiology of bleeding in critically ill patients is complex. Stress-related mucosal injury, impaired mucosal perfusion, systemic inflammation, organ dysfunction, thrombocytopenia, anticoagulant exposure, and coagulopathy may coexist (5). Fibrinolysis may be only one component of the haemostatic disturbance, and inhibition of fibrinolysis alone may be insufficient to prevent progression to clinically important bleeding. Third, the decision to administer TXA in clinical practice is likely influenced by physician concern regarding bleeding severity or risk of progression. This creates a risk of confounding by indication, which may persist despite adjustment using propensity score methods (20).

Our findings are broadly consistent with contemporary evidence questioning the routine use of TXA for gastrointestinal bleeding. Although earlier studies and some meta-analyses suggested potential reductions in rebleeding, large clinical trials and current guideline recommendations have not supported routine TXA administration for UGIB (16, 17). The HALT-IT trial, a large international randomized controlled trial, found that TXA did not reduce death due to gastrointestinal bleeding and was associated with increased venous thromboembolic events (15). Nevertheless, the present study addresses a distinct and previously unanswered clinical question. HALT-IT enrolled patients with significant gastrointestinal bleeding deemed to be at risk of death—a population in which the indication for adjunctive hemostatic therapy, though ultimately not supported by the trial, was at least clinically plausible. In contrast, our cohort comprised critically ill patients whose initial bleeding did not meet established criteria for clinically important hemorrhage. This represents a substantially more common yet therapeutically ambiguous scenario in routine ICU practice: the patient with minor overt bleeding in whom the clinician must decide whether to administer TXA empirically, without clear evidence to guide the decision. Thus, while HALT-IT provides high-level evidence against routine TXA use in major UGIB, the benefit-risk profile in this milder bleeding population has remained uncertain. Our study contributes observational evidence that empirical TXA use in this specific context was not associated with a statistically detectable reduction in bleeding progression, thereby addressing an important evidence gap left by HALT-IT and prior meta-analyses. This population differs from general UGIB cohorts because patients frequently have severe systemic illness, multiple competing causes of haemoglobin decline, high baseline thrombotic risk, and complex medication exposure. The absence of a significant reduction in clinically important bleeding after both PSM and IPTW analysis suggests that TXA is unlikely to have a large protective effect in this setting.

The primary outcome deserves particular consideration. Clinically important GIB was defined using haemoglobin decline, haemodynamic instability, or need for intervention. These criteria are clinically relevant and have been used in studies of stress ulcer-related bleeding in critically ill patients (18). However, in critically ill patients they may be influenced by factors other than gastrointestinal bleeding alone. For example, haemoglobin changes may be affected by fluid resuscitation, phlebotomy, renal replacement therapy, surgery, trauma, or transfusion practice. Haemodynamic instability may also reflect sepsis, shock, sedative exposure, or cardiac dysfunction rather than bleeding progression. Therefore, progression from non-clinically important to clinically important bleeding in ICU patients may reflect both local gastrointestinal haemostasis and overall disease severity. This interpretation is supported by the exploratory regression analyses, in which markers of illness severity, SOFA-2 score, was associated with clinically important bleeding in univariate analysis, whereas TXA use was not. In multivariable analysis, SOFA-2 score remained independently associated with clinically important bleeding, further emphasizing the importance of systemic illness severity.

In this study, TXA was not associated with a significant reduction in transfusion requirement after PSM. In the IPTW analysis, red blood cell transfusion was more frequent in the TXA group. This result should be interpreted cautiously. Because TXA was administered at the discretion of treating physicians, patients receiving TXA may have been perceived as being at higher bleeding risk or having more concerning bleeding manifestations, even if measured baseline variables appeared balanced. Such residual confounding may explain the higher transfusion rate rather than a direct harmful effect of TXA (9, 13, 14). Nevertheless, the absence of a transfusion-sparing effect is clinically important, because reduction in transfusion is one of the major expected benefits of haemostatic therapy.

The lower number of 28-day ventilator-free days in the TXA group also warrants careful interpretation. In the matched cohort, TXA recipients had significantly fewer ventilator-free days (11 (5, 20) vs. 18 (8, 25) days; *P* = 0.019), although total mechanical ventilation duration was not statistically different (9 (5, 14) vs. 9 (3, 20) days; *P* = 0.266). Similar findings were observed in subgroup analyses, but these analyses were exploratory and based on limited sample sizes, and should therefore be interpreted with caution. These associations may reflect residual confounding by disease severity, clinician selection, or unmeasured factors such as respiratory failure severity, bleeding-related aspiration, neurological status, or limitations of care. Given the retrospective design and the exploratory nature of subgroup findings, these results should not be interpreted as evidence that TXA prolongs mechanical ventilation. However, they reinforce the need for caution when using TXA empirically in critically ill patients without clear evidence of benefit.

Thrombotic safety is an important concern when considering TXA in ICU patients, as this population frequently exhibits a prothrombotic baseline state driven by inflammation, immobilization, vascular catheterization, infection, malignancy, surgery, and organ dysfunction (21, 22). In the propensity score-matched cohort, the incidences of deep vein thrombosis (11.7% vs. 16.9%; *P* = 0.256), pulmonary embolism (1.9% vs. 0.6%; *P* = 0.625), and myocardial infarction (5.8% vs. 3.2%; *P* = 0.412) did not differ significantly between groups. However, the IPTW sensitivity analysis yielded divergent associations for deep vein thrombosis (9.7% vs. 17.9%; *P* < 0.001) and myocardial infarction (5.6% vs. 1.9%; *P* = 0.001), while pulmonary embolism remained comparable (1.7% vs. 1.1%; *P* = 0.210). These discrepancies—observed across multiple secondary endpoints including transfusion requirements, ventilator-free days, and mechanical ventilation duration—warrant careful interpretation. Several factors may contribute: the two analytical approaches target different estimands (ATT for PSM vs. ATE for IPTW), PSM restricts the sample to 154 matched pairs thereby reducing statistical power for secondary outcomes, and residual confounding may differ between methods. Moreover, given the multiple secondary outcomes examined, the possibility of type I error cannot be excluded. Notably, the primary outcome of clinically important bleeding and in-hospital mortality demonstrated consistent results across both PSM and IPTW, supporting the robustness of our main conclusions. The secondary outcome findings should therefore be interpreted cautiously and considered exploratory rather than confirmatory. Taken together, the thrombotic safety profile of TXA in this population remains inconclusive, and the absence of a statistically significant increase in thrombotic events in the matched analysis should not be interpreted as definitive evidence of safety.

This study has several clinical implications. First, in critically ill patients with non-clinically important non-variceal UGIB, empirical TXA use should not be considered a substitute for careful clinical assessment, haemodynamic optimization, correction of reversible coagulopathy, acid suppression when appropriate, and timely endoscopic evaluation when bleeding becomes clinically significant (16, 17). Second, the decision to use TXA should be individualized rather than routine, especially in patients with high thrombotic risk or uncertain bleeding activity. Third, future studies should distinguish between clinically important and non-clinically important bleeding, because the benefit-risk profile of haemostatic therapy may differ substantially according to bleeding severity. Finally, the timing of TXA administration relative to bleeding onset may be critical and should be prospectively captured in future research (14, 23, 24).

The present study has several strengths. It focused on a clinically relevant but under-investigated population: critically ill patients with non-clinically important non-variceal UGIB. The sample size was relatively large for this specific clinical scenario, and patients receiving other haemostatic agents were excluded to reduce contamination of the exposure definition. In addition, both PSM and IPTW were used to address baseline differences, and subgroup analyses were performed to explore whether the association between TXA and outcomes differed across clinically relevant patient groups.

Several limitations should be acknowledged. First, this was a single-center retrospective observational study with clinician-driven treatment allocation, which precludes causal inference. Although propensity score-based methods were applied, residual confounding and confounding by indication cannot be fully excluded (25, 26), particularly given the temporal overlap between the exposure window and early outcome development. Second, exact timestamps of TXA administration were not available; only interval times were recorded. A time-dependent exposure analysis therefore could not be reliably performed, and we used a 24-hour landmark analysis instead. In this approach, patients who developed clinically important bleeding, died, were discharged, or were lost to follow-up before the 24-hour landmark were excluded from both groups according to the same rule, and follow-up for the primary outcome began at the 24-hour landmark in both groups. Although this design mitigates immortal time bias and ensures a common start of follow-up, landmark analysis may attenuate effect estimates toward the null and reduce statistical power because early events are excluded. In addition, because this was a retrospective study, the accuracy and completeness of laboratory results and treatment details recorded in the electronic medical record during the period from time zero to the 24-hour landmark could not be fully guaranteed. The exact timing of some tests or treatments may have been imprecise, and some interventions may have been recorded only as having occurred within a certain interval. This could lead to misclassification of baseline covariates and residual confounding. Therefore, residual selection bias related to the exposure-assignment window cannot be entirely excluded, and the results should be regarded as exploratory. Third, TXA administration was not standardized; dosing, timing, and duration were determined by treating physicians, introducing exposure heterogeneity. Fourth, outcome classification may have been affected by the inherent challenges of defining bleeding onset in ICU patients and by the influence of non-bleeding factors (e.g., fluid resuscitation, phlebotomy) on hemoglobin decline and hemodynamic stability. Fifth, all six SOFA-2 organ systems were scored. The brain component was scored using the Glasgow Coma Scale (GCS), and the kidney component using serum creatinine. However, two additive SOFA-2 criteria were completely unavailable in this retrospective dataset (100% missing for all patients): the criterion assigning at least 1 point for delirium requiring pharmacologic treatment, and the urine output criteria for the kidney component (19). These supplementary criteria could not be substituted or imputed because they were not recorded for any patient. Because they are additive rather than primary scoring variables, their omission may have led to a 1-point underestimation in the brain component and a possible underestimation in the kidney component for some patients, rather than a systematic absence of organ dysfunction scoring. No entire organ system was assigned a score of 0 because of missing data. A sensitivity analysis restricted to complete SOFA-2 data was not feasible because these two criteria were entirely unavailable. This limitation should be considered when interpreting severity-adjusted analyses. Sixth, the subgroup and longitudinal analyses were underpowered due to the limited number of primary outcome events (*n* = 21) and informative missingness from death and discharge, respectively; these findings should be considered exploratory. Seventh, although IPTW was used to minimize confounding by indication, covariate balance was not fully achieved for all baseline characteristics despite weight truncation and stabilization, reflecting the inherent limitations of weighting approaches in cohorts with substantial baseline differences (154 vs. 1,660). To mitigate this, we implemented doubly robust estimation by additionally adjusting for covariates with residual imbalance in the outcome regression models. Nevertheless, the possibility of residual confounding cannot be entirely excluded, and these findings should be interpreted with appropriate caution. Finally, the single-center design may limit generalizability to other settings with different patient populations or practice patterns.

## 5 Conclusion

In conclusion, among critically ill patients with non-clinically important non-variceal UGIB, TXA use was not associated with a statistically detectable reduction in the risk of progression to clinically important GIB, nor with significant improvements in secondary outcomes. However, given the limited number of primary outcome events and the wide confidence intervals, a modest clinically meaningful benefit or harm cannot be excluded. These findings should be interpreted as supportive observational evidence rather than definitive proof of treatment futility, and routine empirical use of TXA in this population should be approached with caution. Prospective studies with standardized TXA dosing, precise timing of administration, rigorous bleeding classification, and systematic safety monitoring are needed to clarify whether selected subgroups may benefit from antifibrinolytic therapy.

## Data Availability

The original contributions presented in the study are included in the article/Supplementary Material, further inquiries can be directed to the corresponding authors.
