# Supplementary material for: Tranexamic acid use and progression to clinically important bleeding in critically ill patients with non-clinically important non-variceal upper gastrointestinal bleeding: a retrospective cohort study
Source: Front Med (Lausanne). 2026 Oct 1;13:1940595. doi: 10.3389/fmed.2026.1940595 (PMC13668849; doi:10.3389/fmed.2026.1940595)
Supplement: Supplementary file 1 [file Table1.docx]

**Supplementary Table 1.** Characteristics of TXA group and Non-TXA group patients at ICU admission after IPTW.

| Parameters (mean ± SD) | TXA group (Weighted n ≈ 1302) | Non-TXA group (Weighted n ≈ 1822) | P-value | SMD |
| --- | --- | --- | --- | --- |
| Age, y | 55.20 ± 15.72 | 54.37 ± 16.90 | 0.162 | 0.051 |
| Male n% | 969 (74.4%) | 1263 (69.3%) | 0.002 | 0.112 |
| APACHE Ⅱ | 20.82 ± 6.72 | 18.50 ± 8.66 | ＜0.001 | 0.204 |
| Type of admission |  |  | ＜0.001 | 0.198 |
| Medical n% | 732 (56.2%) | 989 (54.3%) |  |  |
| Surgical n% | 571 (43.8%) | 833 (45.7%) |  |  |
| Complication |  |  |  |  |
| Diabetes n% | 129 (9.9%) | 199 (10.9%) | 0.362 | -0.032 |
| CKD n% | 50 (3.8%) | 83 (4.6%) | 0.327 | -0.036 |
| Acute coronary syndrome n% | 73 (5.6%) | 172 (9.4%) | ＜0.001 | -0.145 |
| Severe pneumonia n% | 617 (47.4%) | 980 (53.8%) | ＜0.001 | -0.129 |
| Burn victim n% | 0 | 8 (0.4%) | 0.024 | -0.093 |
| Cancer n% | 211 (16.2%) | 200 (11.0%) | ＜0.001 | 0.153 |
| Neurological disorders n% | 281 (21.6%) | 535 (29.4%) | ＜0.001 | -0.181 |
| Severe acute pancreatitis n% | 222 (17.0%) | 225 (12.3%) | ＜0.001 | 1.322 |
| Trauma n% | 166 (12.7%) | 248 (13.6%) | 0.480 | -0.026 |

“Weighted n” represents pseudo-sample sizes (sum of weights) after IPTW and should not be interpreted as actual patient counts. Actual patient numbers were 154 in the TXA group and 1,660 in the non-TXA group. CKD: Chronic Kidney Disease.

**Supplementary Table 2.** Characteristics of TXA group and Non-TXA group patients on the day of 24-hour landmark after non-clinically important NVUGIB after IPTW.

| Parameters (mean ± SD) / median (IQR) | TXA group (Weighted n ≈ 1302) | Non-TXA group (Weighted n ≈ 1822) | P-value | SMD |
| --- | --- | --- | --- | --- |
| Time to Bleeding, d | 9 (5, 14) | 7 (4, 12) | 0.003 | 0.106 |
| SOFA-2 score | 5.91 ± 3.86 | 5.81 ± 3.73 | 0.960 | 0.028 |
| HGB, g/L | 90.50 ± 17.83 | 93.26 ± 20.04 | ＜0.001 | -0.146 |
| PLT, 10^9^/L | 192.98 ± 140.69 | 189.27 ± 134.27 | 0.459 | 0.027 |
| INR | 1.26 ± 0.45 | 1.26 ± 0.41 | 0.995 | ＜0.001 |
| APPT, sec | 35.80 ± 8.02 | 34.76 ± 10.37 | 0.001 | 0.113 |
| PT, sec | 14.05 ± 3.26 | 13.95 ± 5.06 | 0.538 | 0.199 |
| CREA, umol/L | 101.36 ± 94.81 | 94.59 ± 91.57 | 0.046 | 0.073 |
| UREA, umol/L | 12.82 ± 8.32 | 10.96 ± 9.74 | 0.014 | 0.201 |
| ALB, g/L | 33.59 ± 5.89 | 33.58 ± 6.04 | ＜0.001 | 0.002 |
| TBIL, umol/L | 38.59 ± 54.57 | 31.53 ± 50.39 | ＜0.001 | 0.134 |
| DBIL, umol/L | 28.53 ± 46.37 | 20.87 ± 39.00 | ＜0.001 | 0.179 |
| CRP, mg/L | 109.82 ± 101.57 | 104.28 ± 92.55 | 0.038 | 0.057 |
| PCT, ng/mL | 4.00 ± 7.04 | 3.20 ± 9.86 | 0.008 | 0.094 |
| WBC, 10^9^/L | 11.38 ± 5.57 | 11.52 ± 6.76 | 0.119 | -0.024 |
| LAC, mmol/L | 1.85 ± 1.38 | 1.92 ± 1.74 | 0.215 | -0.044 |
| Treatment |  |  |  |  |
| Vasoactive-inotropic drugs n% | 374 (28.7%) | 575 (31.6%) | 0.090 | 0.062 |
| Maximum infusion rate of norepinephrine, μg/kg/min | 0 (0, 1.36) | 0 (0, 0.06) | 0.644 | 0.017 |
| Mechanical ventilation n% | 752 (57.8%) | 1024 (56.2%) | 0.396 | 0.030 |
| Proton pump inhibitors n% | 536 (41.2%) | 937 (51.5%) | ＜0.001 | -0.206 |
| Somatostatin n% | 359 (27.6%) | 384 (21.1%) | ＜0.001 | 0.152 |
| Anticoagulant drugs n% | 184 (14.1%) | 263 (14.4%) | 0.800 | -0.011 |
| Red blood cell suspension transfusion n% | 24 (1.8%) | 15 (0.8%) | 0.018 | 0.090 |
| Plasma transfusion n% | 136 (10.4%) | 142 (7.8%) | 0.010 | 0.092 |
| Enteral Nutrition n% | 290 (23.3%) | 452 (24.8%) | 0.099 | -0.060 |

Time to Bleeding: The number of days from admission to the ICU to the day of 24-hour landmark after non-clinically important NVUGIB. SOFA-2: Sequential Organ Failure Assessment-2; APACHE II: Acute Physiology and Chronic Health Evaluation II; HGB: Hemoglobin; RBC: Red Blood Cell; PLT: Platelet; INR: International Normalized Ratio; PT: Prothrombin Time; APTT: Activated Partial Thromboplastin Time; CREA: Creatinine; UREA: Urea; ALB: Albumin; TBIL: Total Bilirubin; DBIL: Direct Bilirubin; IBIL: Indirect Bilirubin; AST: Aspartate Aminotransferase; ALT: Alanine Aminotransferase; CRP: C-Reactive Protein; PCT: Procalcitonin; WBC: White Blood Cell; LAC: Lactate; PPI: Proton Pump Inhibitors. “Weighted n” represents pseudo-sample sizes (sum of weights) after IPTW and should not be interpreted as actual patient counts. Actual patient numbers were 154 in the TXA group and 1,660 in the non-TXA group.

Supplementary Figures 1A–1G: The Changing trend of APTT, PT, FB, ATIII, WBC, PCT and ALB for the study population during the 3 days before and 12 days after the bleeding event, record the average value every 3 days.

(A) Changing trend of APTT from Day -3 to Day 12. (B) Changing trend of PT from Day -3 to Day 12. (C) Changing trend of FB from Day -3 to Day 12. (D) Changing trend of AT-III from Day -3 to Day 12. (E) Changing trend of PCT from Day -3 to Day 12. (F) Changing trend of WBC from Day -3 to Day 12. (G) Changing trend of ALB from Day -3 to Day 12. Day 0: 0–24 hours after the index UGIB episode (time zero to the 24-hour landmark). PT: Prothrombin Time; APTT: Activated Partial Thromboplastin Time; FB: Fibrinogen; AT-III: Antithrombin III; PCT: Procalcitonin; WBC: White Blood Cell; ALB: Albumin; ***** *p*<0.05, ******* *p*<0.001, ns: non-significant.

**Supplementary Table 3.** Characteristics of TXA group and Non-TXA group patients at ICU admission: Subgroup analysis of patients with SOFA-2 score ≥ 6.

| Parameters (mean ± SD) | TXA group (n=73) | Non-TXA group (n=64) | P-value |
| --- | --- | --- | --- |
| Age, y | 53.82 ± 16.86 | 52.92 ± 17.88 | 0.763 |
| Male n% | 55 (75.3%) | 51 (79.7%) | 0.544 |
| APACHE Ⅱ | 21.75 ± 6.73 | 20.28 ± 8.80 | 0.279 |
| Type of admission |  |  | 0.415 |
| Medical n% | 46 (63.0%) | 35 (54.7%) |  |
| Surgical n% | 27 (37.0%) | 29 (51.8%) |  |
| Complication |  |  |  |
| Diabetes n% | 12 (16.4%) | 6 (9.4%) | 0.333 |
| CKD n% | 5 (6.8%) | 3 (4.7%) | 0.590 |
| Acute coronary syndrome n% | 5 (6.8%) | 5 (7.8%) | >0.999 |
| Severe pneumonia n% | 37 (50.7%) | 38 (59.4%) | 0.397 |
| Burn victim n% | 0 | 0 |  |
| Cancer n% | 10 (13.7%) | 6 (9.4%) | 0.603 |
| Neurological disorders n% | 14 (19.2%) | 20 (31.3%) | 0.152 |
| Severe acute pancreatitis n% | 18 (24.7%) | 12 (18.8%) | 0.531 |
| Trauma n% | 8 (11.0%) | 9 (14.1%) | 0.772 |

CKD: Chronic Kidney Disease.

**Supplementary Table 4.** Characteristics of TXA group and Non-TXA group patients on the day of 24-hour landmark after non-clinically important NVUGIB: Subgroup analysis of patients with SOFA-2 score ≥ 6.

| Parameters (mean ± SD) / median (IQR) | TXA group (n=73) | Non-TXA group (n=64) | P-value |
| --- | --- | --- | --- |
| Time to Bleeding, d | 9 (5, 14) | 8 (4, 13) | 0.881 |
| SOFA-2 score | 9.03 ± 2.97 | 10.00 ± 3.29 | 0.073 |
| HGB, g/L | 87.32 ± 19.69 | 85.44 ± 16.77 | 0.548 |
| PLT, 10^9^/L | 128.05 ± 112.30 | 129.70 ± 125.32 | 0.936 |
| INR | 1.40 ± 0.72 | 1.35 ± 0.38 | 0.615 |
| APPT, sec | 38.50 ± 9.81 | 42.87 ± 22.79 | 0.159 |
| PT, sec | 14.91 ± 4.26 | 15.27 ± 3.94 | 0.601 |
| CREA, umol/L | 140.41 ± 131.69 | 146.41 ± 130.33 | 0.774 |
| UREA, umol/L | 15.52 ± 9.75 | 17.89 ± 15.15 | 0.287 |
| ALB, g/L | 32.97 ± 6.42 | 32.60 ± 5.91 | 0.729 |
| TBIL, umol/L | 61.77 ± 71.38 | 58.75 ± 85.16 | 0.824 |
| DBIL, umol/L | 46.26 ± 59.40 | 43.70 ± 72.78 | 0.731 |
| CRP, mg/L | 123.51 ± 110.85 | 109.12 ± 85.02 | 0.392 |
| PCT, ng/mL | 6.26 ± 10.05 | 6.63 ± 13.51 | 0.858 |
| WBC, 10^9^/L | 11.63 ± 6.68 | 12.41 ± 7.83 | 0.536 |
| LAC, mmol/L | 2.32 ± 1.90 | 2.32 ± 1.76 | 0.982 |
| Treatment |  |  |  |
| Vasoactive-inotropic drugs n% | 37 (50.7%) | 35 (54.7%) | 0.767 |
| Maximum infusion rate of norepinephrine, μg/kg/min | 0.05 (0, 0.3) | 0.1 (0, 0.4) | 0.353 |
| Mechanical ventilation n% | 57 (78.1%) | 51 (79.7%) | 0.818 |
| Proton pump inhibitors n% | 28 (38.4%) | 22 (34.4%) | 0.760 |
| Somatostatin n% | 24 (32.9%) | 21 (32.8%) | >0.999 |
| Anticoagulant drugs n% | 9 (12.3%) | 8 (12.5%) | >0.999 |
| Red blood cell suspension transfusion n% | 2 (2.7%) | 1 (1.6%) | >0.999 |
| Plasma transfusion n% | 12 (16.4%) | 8 (12.5%) | 0.683 |
| Enteral Nutrition n% | 13 (17.8%) | 9 (14.1%) | 0.717 |

Time to Bleeding: The number of days from admission to the ICU to the day of 24-hour landmark after non-clinically important NVUGIB. SOFA-2: Sequential Organ Failure Assessment-2; HGB: Hemoglobin; RBC: Red Blood Cell; PLT: Platelet; INR: International Normalized Ratio; PT: Prothrombin Time; APTT: Activated Partial Thromboplastin Time; CREA: Creatinine; UREA: Urea; ALB: Albumin; TBIL: Total Bilirubin; DBIL: Direct Bilirubin; IBIL: Indirect Bilirubin; AST: Aspartate Aminotransferase; ALT: Alanine Aminotransferase; CRP: C-Reactive Protein; PCT: Procalcitonin; WBC: White Blood Cell; LAC: Lactate; PPI: Proton Pump Inhibitors.

**Supplementary Table 5.** Characteristics of TXA group and Non-TXA group patients at ICU admission: Subgroup analysis of patients using anticoagulants.

| Parameters (mean ± SD) | TXA group (n=21) | Non-TXA group (n=18) | P-value |
| --- | --- | --- | --- |
| Age, y | 60.19 ± 18.94 | 54.78 ± 15.17 | 0.328 |
| Male n% | 17 (81.0%) | 11 (61.1%) | 0.310 |
| APACHE Ⅱ | 22.10 ± 7.96 | 22.72 ± 8.38 | 0.813 |
| Type of admission |  |  | 0.307 |
| Medical n% | 12 (57.1%) | 24 (77.8%) |  |
| Surgical n% | 9 (42.9%) | 4 (22.2%) |  |
| Complication |  |  |  |
| Diabetes n% | 5 (8.6%) | 5 (9.8%) | >0.999 |
| CKD n% | 4 (6.9%) | 2 (3.9%) | 0.683 |
| Acute coronary syndrome n% | 0 | 1 (5.6%) | 0.462 |
| Severe pneumonia n% | 28 (48.3%) | 28 (54.9%) | 0.618 |
| Burn victim n% | 0 | 0 |  |
| Cancer n% | 5 (8.6%) | 4 (7.8%) | >0.999 |
| Neurological disorders n% | 10 (17.2%) | 15 (29.4%) | 0.201 |
| Severe acute pancreatitis n% | 15 (25.9%) | 6 (11.8%) | 0.106 |
| Trauma n% | 7 (12.1%) | 6 (11.8%) | >0.999 |

CKD: Chronic Kidney Disease.

**Supplementary Table 6.** Characteristics of TXA group and Non-TXA group patients on the day of 24-hour landmark after non-clinically important NVUGIB: Subgroup analysis of patients using anticoagulants.

| Parameters (mean ± SD) / median (IQR) | TXA group (n=26) | Non-TXA group (n=34) | P-value |
| --- | --- | --- | --- |
| Time to Bleeding, d | 8 (5, 13) | 8 (5, 14) | 0.756 |
| SOFA-2 score | 5.67 ± 4.21 | 5.78 ± 4.05 | 0.934 |
| HGB, g/L | 91.33 ± 16.95 | 83.50 ± 11.06 | 0.193 |
| PLT, 10^9^/L | 154.38 ± 77.63 | 218.17 ± 188.19 | 0.669 |
| INR | 1.18 ± 0.20 | 1.27 ± 0.22 | 0.214 |
| APPT, sec | 34.25 ± 6.57 | 35.46 ± 11.09 | 0.690 |
| PT, sec | 13.33 ± 2.08 | 14.12 ± 2.45 | 0.287 |
| CREA, umol/L | 101.48 ± 80.26 | 97.11 ± 50.67 | 0.838 |
| UREA, umol/L | 13.26 ± 8.76 | 13.76 ± 11.94 | 0.884 |
| ALB, g/L | 32.85 ± 3.88 | 33.32 ± 2.95 | 0.673 |
| TBIL, umol/L | 47.19 ± 64.28 | 23.11 ± 24.71 | 0.125 |
| DBIL, umol/L | 37.03 ± 61.43 | 17.23 ± 24.07 | 0.185 |
| CRP, mg/L | 121.72 ± 92.72 | 103.99 ± 92.45 | 0.272 |
| PCT, ng/mL | 3.18 ± 3.54 | 15.78 ± 28.77 | 0.557 |
| WBC, 10^9^/L | 9.76 ± 5.80 | 9.66 ± 4.62 | 0.953 |
| LAC, mmol/L | 1.55 ± 0.55 | 2.52 ± 2.15 | 0.078 |
| Treatment |  |  |  |
| Vasoactive-inotropic drugs n% | 16 (27.6%) | 16 (31.4%) | 0.824 |
| Maximum infusion rate of norepinephrine, μg/kg/min | 0 (0, 0.03) | 0 (0, 0.08) | 0.303 |
| Mechanical ventilation n% | 32 (55.2%) | 29 (56.9%) | >0.999 |
| Proton pump inhibitors n% | 10 (47.6%) | 3 (16.7%) | 0.088 |
| Somatostatin n% | 17 (29.3%) | 16 (31.4%) | 0.9 |
| Red blood cell suspension transfusion n% | 1 (4.8%) | 0 | >0.999 |
| Plasma transfusion n% | 8 (13.8%) | 5 (9.8%) | 0.730 |
| Enteral Nutrition n% | 7 (33.3%) | 6 (33.3%) | >0.999 |

Time to Bleeding: The number of days from admission to the ICU to the day of 24-hour landmark after non-clinically important NVUGIB. SOFA-2: Sequential Organ Failure Assessment-2; HGB: Hemoglobin; RBC: Red Blood Cell; PLT: Platelet; INR: International Normalized Ratio; PT: Prothrombin Time; APTT: Activated Partial Thromboplastin Time; CREA: Creatinine; UREA: Urea; ALB: Albumin; TBIL: Total Bilirubin; DBIL: Direct Bilirubin; IBIL: Indirect Bilirubin; AST: Aspartate Aminotransferase; ALT: Alanine Aminotransferase; CRP: C-Reactive Protein; PCT: Procalcitonin; WBC: White Blood Cell; LAC: Lactate.

**Supplementary Table 7.** Characteristics of TXA group and Non-TXA group patients at ICU admission: Subgroup analysis of patients with sepsis.

| Parameters (mean ± SD) | TXA group (n=88) | Non-TXA group (n=89) | P-value |
| --- | --- | --- | --- |
| Age, y | 56.34 ± 15.63 | 54.03 ± 17.05 | 0.349 |
| Male n% | 63 (71.6%) | 70 (78.7%) | 0.277 |
| APACHE Ⅱ | 22.49 ± 7.02 | 21.67 ± 8.84 | 0.498 |
| Type of admission |  |  | 0.391 |
| Medical n% | 56 (63.6%) | 50 (56.2%) |  |
| Surgical n% | 32 (36.4%) | 39 (43.8%) |  |
| Complication |  |  |  |
| Diabetes n% | 8 (9.1%) | 9 (10.1%) | >0.999 |
| CKD n% | 3 (3.4%) | 4 (4.5%) | >0.999 |
| Acute coronary syndrome n% | 6 (6.8%) | 9 (10.1%) | 0.605 |
| Severe pneumonia n% | 37 (42.0%) | 47 (52.8%) | 0.199 |
| Burn victim n% | 0 | 0 |  |
| Cancer n% | 17 (19.3%) | 11 (12.4%) | 0.288 |
| Neurological disorders n% | 12 (13.6%) | 17 (19.1%) | 0.436 |
| Severe acute pancreatitis n% | 18 (20.5%) | 14 (15.7%) | 0.534 |
| Trauma n% | 9 (10.2%) | 8 (9.0%) | 0.980 |

CKD: Chronic Kidney Disease.

**Supplementary Table 8.** Characteristics of TXA group and Non-TXA group patients on the day of 24-hour landmark after non-clinically important NVUGIB: Subgroup analysis of patients with sepsis.

| Parameters (mean ± SD) / median (IQR) | TXA group (n=88) | Non-TXA group (n=89) | P-value |
| --- | --- | --- | --- |
| Time to Bleeding, d | 6 (4, 14) | 7 (4, 12) | 0.972 |
| SOFA-2 score | 5.89 ± 4.04 | 5.89 ± 4.21 | 0.998 |
| HGB, g/L | 90.38 ± 18.38 | 90.67 ± 20.45 | 0.919 |
| PLT, 10^9^/L | 200.80 ± 146.01 | 201.73 ± 153.31 | 0.967 |
| INR | 1.24 ± 0.37 | 1.25 ± 0.35 | 0.888 |
| APPT, sec | 35.66 ± 8.27 | 36.87 ± 19.46 | 0.590 |
| PT, sec | 14.10 ± 3.93 | 14.12 ± 3.56 | 0.971 |
| CREA, umol/L | 112.58 ± 123.93 | 115.47 ± 138.56 | 0.884 |
| UREA, umol/L | 12.69 ± 8.54 | 11.34 ± 10.98 | 0.360 |
| ALB, g/L | 33.39 ± 6.37 | 33.56 ± 5.85 | 0.851 |
| TBIL, umol/L | 42.75 ± 62.73 | 36.61 ± 66.96 | 0.530 |
| DBIL, umol/L | 30.40 ± 50.91 | 20.01 ± 56.81 | 0.590 |
| CRP, mg/L | 105.33 ± 101.34 | 99.74 ± 92.61 | 0.702 |
| PCT, ng/mL | 4.64 ± 9.08 | 2.79 ± 5.19 | 0.100 |
| WBC, 10^9^/L | 11.35 ± 5.45 | 12.24 ± 7.09 | 0.492 |
| LAC, mmol/L | 1.86 ± 1.15 | 1.86 ± 1.38 | 0.987 |
| Treatment |  |  |  |
| Vasoactive-inotropic drugs n% | 24 (27.3%) | 21 (23.6%) | 0.697 |
| Maximum infusion rate of norepinephrine, μg/kg/min | 0 (0, 0.09) | 0 (0, 0) | 0.753 |
| Mechanical ventilation n% | 50 (56.8%) | 46 (51.7%) | 0.493 |
| Proton pump inhibitors n% | 29 (33.0%) | 33 (37.1%) | 0.676 |
| Somatostatin n% | 25 (28.4%) | 25 (28.1%) | >0.999 |
| Red blood cell suspension transfusion n% | 2 (2.3%) | 1 (1.1%) | 0.621 |
| Plasma transfusion n% | 11 (12.5%) | 6 (6.7%) | 0.296 |
| Anticoagulant drugs n% | 13 (14.8%) | 12 (13.5%) | 0.976 |
| Enteral Nutrition n% | 19 (21.6%) | 20 (22.5%) | >0.999 |

Time to Bleeding: The number of days from admission to the ICU to the day of 24-hour landmark after non-clinically important NVUGIB. SOFA-2: Sequential Organ Failure Assessment-2; HGB: Hemoglobin; RBC: Red Blood Cell; PLT: Platelet; INR: International Normalized Ratio; PT: Prothrombin Time; APTT: Activated Partial Thromboplastin Time; CREA: Creatinine; UREA: Urea; ALB: Albumin; TBIL: Total Bilirubin; DBIL: Direct Bilirubin; IBIL: Indirect Bilirubin; AST: Aspartate Aminotransferase; ALT: Alanine Aminotransferase; CRP: C-Reactive Protein; PCT: Procalcitonin; WBC: White Blood Cell; LAC: Lactate.

**Supplementary Table 9.** Characteristics of TXA group and Non-TXA group patients at ICU admission: Subgroup analysis of patients with a primary neurological diagnosis.

| Parameters (mean ± SD) | TXA group (n=29) | Non-TXA group (n=36) | P-value |
| --- | --- | --- | --- |
| Age, y | 54.38 ± 14.91 | 54.53 ± 16.79 | 0.970 |
| Male n% | 23 (79.3%) | 24 (66.7%) | 0.393 |
| APACHE Ⅱ | 21.28 ± 6.99 | 22.44 ± 8.20 | 0.538 |
| Type of admission |  |  | >0.999 |
| Medical n% | 17 (58.6%) | 20 (55.6%) |  |
| Surgical n% | 12 (41.4%) | 16 (44.4%) |  |
| Complication |  |  |  |
| Diabetes n% | 6 (20.7%) | 5 (13.9%) | 0.693 |
| CKD n% | 1 (3.4%) | 2 (5.6%) | >0.999 |
| Acute coronary syndrome n% | 1 (3.4%) | 1 (2.8%) | >0.999 |
| Severe pneumonia n% | 14 (48.3%) | 18 (50.0%) | >0.999 |
| Cancer n% | 3 (10.3%) | 2 (5.6%) | 0.801 |
| Severe acute pancreatitis n% | 0 | 0 | - |
| Trauma n% | 1 (3.4%) | 5 (13.9%) | 0310 |

CKD: Chronic Kidney Disease.

**Supplementary Table 10.** Characteristics of TXA group and Non-TXA group patients on the day of 24-hour landmark after non-clinically important NVUGIB: Subgroup analysis of patients with a primary neurological diagnosis.

| Parameters (mean ± SD) / median (IQR) | TXA group (n=29) | Non-TXA group (n=36) | P-value |
| --- | --- | --- | --- |
| Time to Bleeding, d | 12 (7, 18) | 9 (5, 13) | 0.056 |
| SOFA-2 score | 5.72 ± 3.79 | 6.83 ± 4.72 | 0.297 |
| HGB, g/L | 92.55 ± 17.05 | 85.03 ± 15.34 | 0.070 |
| PLT, 10^9^/L | 175.14 ± 99.44 | 150.11 ± 97.77 | 0.314 |
| INR | 1.27 ± 0.26 | 1.28 ± 0.29 | 0.892 |
| APPT, sec | 37.03 ± 8.19 | 37.81 ± 10.71 | 0.741 |
| PT, sec | 14.36 ± 2.71 | 14.47 ± 2.99 | 0.872 |
| CREA, umol/L | 85.93 ± 45.47 | 120.19 ± 150.95 | 0.204 |
| UREA, umol/L | 12.46 ± 7.36 | 15.58 ± 13.26 | 0.512 |
| ALB, g/L | 33.41 ± 5.76 | 32.24 ± 5.08 | 0.394 |
| TBIL, umol/L | 42.21 ± 55.27 | 34.05 ± 47.48 | 0.532 |
| DBIL, umol/L | 34.59 ± 53.62 | 24.48 ± 40.77 | 0.406 |
| CRP, mg/L | 132.32 ± 113.33 | 100.06 ± 92.26 | 0.221 |
| PCT, ng/mL | 3.44 ± 4.98 | 8.61 ± 17.35 | 0.096 |
| WBC, 10^9^/L | 10.15 ± 4.67 | 10.10 ± 5.89 | 0.967 |
| LAC, mmol/L | 1.95 ± 2.17 | 2.29 ± 1.95 | 0.517 |
| Treatment |  |  |  |
| Vasoactive-inotropic drugs n% | 8 (27.6%) | 11 (30.6%) | >0.999 |
| Maximum infusion rate of norepinephrine, μg/kg/min | 0 (0, 0.17) | 0 (0, 0.20) | 0.291 |
| Mechanical ventilation n% | 16 (55.2%) | 25 (69.4%) | 0.354 |
| Proton pump inhibitors n% | 10 (34.5%) | 15 (41.7%) | 0.737 |
| Somatostatin n% | 5 (17.2%) | 10 (27.8%) | 0.480 |
| Red blood cell suspension transfusion n% | 1 (3.4%) | 1 (2.8%) | >0.999 |
| Plasma transfusion n% | 4 (13.8%) | 46 (11.1%) | >0.999 |
| Anticoagulant drugs n% | 6 (20.7%) | 5 (13.9%) | 0.693 |
| Enteral Nutrition n% | 11 (37.9%) | 7 (19.4%) | 0.169 |

Time to Bleeding: The number of days from admission to the ICU to the day of 24-hour landmark after non-clinically important NVUGIB. SOFA-2: Sequential Organ Failure Assessment-2; HGB: Hemoglobin; RBC: Red Blood Cell; PLT: Platelet; INR: International Normalized Ratio; PT: Prothrombin Time; APTT: Activated Partial Thromboplastin Time; CREA: Creatinine; UREA: Urea; ALB: Albumin; TBIL: Total Bilirubin; DBIL: Direct Bilirubin; IBIL: Indirect Bilirubin; AST: Aspartate Aminotransferase; ALT: Alanine Aminotransferase; CRP: C-Reactive Protein; PCT: Procalcitonin; WBC: White Blood Cell; LAC: Lactate.

**Supplementary Table 11.** Characteristics of TXA group and Non-TXA group patients at ICU admission: Subgroup analysis of patients with shock.

| Parameters (mean ± SD) | TXA group (n=72) | Non-TXA group (n=71) | P-value |
| --- | --- | --- | --- |
| Age, y | 55.87 ± 15.05 | 52.96 ± 17.71 | 0.156 |
| Male n% | 53 (74.6%) | 54 (75.0%) | >0.999 |
| APACHE Ⅱ | 23.00 ± 6.87 | 21.75 ± 9.20 | 0.359 |
| Type of admission |  |  | 0.525 |
| Medical n% | 48 (67.6%) | 44 (61.1%) |  |
| Surgical n% | 23 (32.4%) | 28 (38.9%) |  |
| Complication |  |  |  |
| Diabetes n% | 10 (14.1%) | 7 (9.7%) | 0.584 |
| CKD n% | 1 (1.4%) | 2 (2.8%) | >0.999 |
| Acute coronary syndrome n% | 6 (8.5%) | 5 (6.9%) | 0.981 |
| Severe pneumonia n% | 34 (47.9%) | 36 (50.0%) | 0.932 |
| Burn victim n% | 0 | 0 | - |
| Cancer n% | 14 (19.7%) | 6 (8.3%) | 0.085 |
| Neurological disorders n% | 12 (16.9%) | 19 (26.4%) | 0.241 |
| Severe acute pancreatitis n% | 16 (22.5%) | 14 (19.4%) | 0.804 |
| Trauma n% | 7 (9.9%) | 6 (8.3%) | 0.979 |

CKD: Chronic Kidney Disease.

**Supplementary Table 12.** Characteristics of TXA group and Non-TXA group patients on the day of 24-hour landmark after non-clinically important NVUGIB: Subgroup analysis of patients with shock.

| Parameters (mean ± SD) | TXA group (n=71) | Non-TXA group (n=72) | P-value |
| --- | --- | --- | --- |
| Time to Bleeding, d | 6 (4, 14) | 7 (4, 12) | 0.195 |
| SOFA-2 score | 6.01 ± 3.91 | 5.35 ± 3.72 | 0.298 |
| HGB, g/L | 92.56 ± 18.47 | 90.29 ± 18.41 | 0.463 |
| PLT, 10^9^/L | 187.38 ± 134.12 | 215.38 ± 159.54 | 0.258 |
| INR | 1.25 ± 0.40 | 1.23 ± 0.35 | 0.792 |
| APPT, sec | 35.39 ± 6.35 | 35.26 ± 19.23 | 0.959 |
| PT, sec | 14.28 ± 4.27 | 13.89 ± 3.61 | 0.560 |
| CREA, umol/L | 106.31 ± 82.20 | 100.19 ± 112.49 | 0.711 |
| UREA, umol/L | 13.19 ± 8.78 | 10.49 ± 9.98 | 0.088 |
| ALB, g/L | 34.18 ± 6.45 | 34.93 ± 5.30 | 0.451 |
| TBIL, umol/L | 51.83 ± 71.57 | 27.86 ± 29.75 | 0.056 |
| DBIL, umol/L | 43.10 ± 69.95 | 19.88 ± 25.73 | 0.018 |
| CRP, mg/L | 105.85 ± 101.47 | 104.66 ± 88.81 | 0.860 |
| PCT, ng/mL | 5.27 ± 9.85 | 2.88 ± 6.22 | 0.086 |
| WBC, 10^9^/L | 11.70 ± 5.74 | 11.87 ± 6.64 | 0.875 |
| LAC, mmol/L | 1.80 ± 1.10 | 1.93 ± 1.37 | 0.525 |
| Treatment |  |  |  |
| Vasoactive-inotropic drugs n% | 20 (28.2%) | 14 (19.4%) | 0.304 |
| Maximum infusion rate of norepinephrine, μg/kg/min | 0 (0, 0.10) | 0 (0, 0) | 0.895 |
| Mechanical ventilation n% | 37 (52.1%) | 39 (54.2%) | 0.937 |
| Proton pump inhibitors n% | 21 (29.6%) | 19 (26.4%) | 0.812 |
| Somatostatin n% | 20 (28.2%) | 19 (26.4%) | 0.959 |
| Red blood cell suspension transfusion n% | 2 (2.8%) | 1 (1.4%) | 0.990 |
| Plasma transfusion n% | 9 (12.7%) | 3 (4.2%) | 0.078 |
| Anticoagulant drugs n% | 9 (12.7%) | 7 (9.7%) | 0.768 |
| Enteral Nutrition n% | 17 (23.9%) | 14 (19.4%) | 0.653 |

Time to Bleeding: The number of days from admission to the ICU to the day of 24-hour landmark after non-clinically important NVUGIB. SOFA-2: Sequential Organ Failure Assessment-2; HGB: Hemoglobin; RBC: Red Blood Cell; PLT: Platelet; INR: International Normalized Ratio; PT: Prothrombin Time; APTT: Activated Partial Thromboplastin Time; CREA: Creatinine; UREA: Urea; ALB: Albumin; TBIL: Total Bilirubin; DBIL: Direct Bilirubin; IBIL: Indirect Bilirubin; AST: Aspartate Aminotransferase; ALT: Alanine Aminotransferase; CRP: C-Reactive Protein; PCT: Procalcitonin; WBC: White Blood Cell; LAC: Lactate.

**Supplementary Table 13.** Characteristics of TXA group and Non-TXA group patients at ICU admission: Subgroup analysis of patients with acute severe pancreatitis.

| Parameters (mean ± SD) | TXA group (n=35) | Non-TXA group (n=29) | P-value |
| --- | --- | --- | --- |
| Age, y | 52.43 ± 14.78 | 56.90 ± 18.51 | 0.290 |
| Male n% | 28 (80.0%) | 18 (62.1%) | 0.192 |
| APACHE Ⅱ | 21.51 ± 8.29 | 19.72 ± 7.25 | 0.219 |
| Type of admission |  |  | 0.165 |
| Medical n% | 24 (68.6%) | 14 (48.3%) |  |
| Surgical n% | 11 (31.4%) | 15 (51.7%) |  |
| Complication |  |  |  |
| Diabetes n% | 3 (8.6%) | 3 (10.3%) | >0.999 |
| CKD n% | 0 | 0 | - |
| Acute coronary syndrome n% | 1 (2.9%) | 0 | >0.999 |
| Severe pneumonia n% | 19 (54.3%) | 21 (72.4%) | 0.213 |

CKD: Chronic Kidney Disease.

**Supplementary Table 14.** Characteristics of TXA group and Non-TXA group patients on the day of 24-hour landmark after non-clinically important NVUGIB: Subgroup analysis of patients with acute severe pancreatitis.

| Parameters (mean ± SD) | TXA group (n=35) | Non-TXA group (n=29) | P-value |
| --- | --- | --- | --- |
| Time to Bleeding, d | 6 (4, 12) | 7 (4, 14) | 0.407 |
| SOFA-2 score | 6.06 ± 3.88 | 6.17 ± 4.41 | 0.978 |
| HGB, g/L | 87.86 ± 18.95 | 88.83 ± 16.27 | 0.826 |
| PLT, 10^9^/L | 192.34 ± 171.06 | 164.14 ± 132.26 | 0.460 |
| INR | 1.45 ± 1.02 | 1.23 ± 0.29 | 0.222 |
| APPT, sec | 37.42 ± 11.44 | 35.61 ± 18.13 | 0.516 |
| PT, sec | 14.36 ± 5.47 | 13.60 ± 3.36 | 0.498 |
| CREA, umol/L | 124.17 ± 156.80 | 95.97 ± 124.75 | 0.426 |
| UREA, umol/L | 13.31 ± 6.97 | 12.63 ± 16.94 | 0.842 |
| ALB, g/L | 34.83 ± 6.50 | 34.16 ± 5.86 | 0.662 |
| TBIL, umol/L | 51.66 ± 68.31 | 38.38 ± 72.53 | 0.457 |
| DBIL, umol/L | 30.59 ± 45.13 | 26.62 ± 64.08 | 0.780 |
| CRP, mg/L | 105.45 ± 91.64 | 92.40 ± 95.43 | 0.581 |
| PCT, ng/mL | 3.99 ± 7.10 | 3.25 ± 6.69 | 0.671 |
| WBC, 10^9^/L | 11.52 ± 6.94 | 12.50 ± 7.35 | 0.591 |
| LAC, mmol/L | 2.41 ± 1.82 | 1.99 ± 1.11 | 0.252 |
| Treatment |  |  |  |
| Vasoactive-inotropic drugs n% | 8 (22.9%) | 7 (24.1%) | >0.999 |
| Maximum infusion rate of norepinephrine, μg/kg/min | 0 (0, 0) | 0 (0, 0.15) | 0.844 |
| Mechanical ventilation n% | 19 (54.3%) | 20 (69.0%) | 0.347 |
| Proton pump inhibitors n% | 15 (42.9%) | 6 (20.7%) | 0.107 |
| Somatostatin n% | 10 (28.6%) | 9 (31.0%) | >0.999 |
| Anticoagulant drugs n% | 3 (8.6%) | 3 (10.3%) | >0.999 |
| Red blood cell suspension transfusion n% | 1 (2.9%) | 0 | >0.999 |
| Plasma transfusion n% | 4 (11.4%) | 1 (3.4%) | 0.366 |
| Enteral Nutrition n% | 7 (20.0%) | 7 (24.1%) | 0.924 |

Time to Bleeding: The number of days from admission to the ICU to the day of 24-hour landmark after non-clinically important NVUGIB. SOFA-2: Sequential Organ Failure Assessment-2; HGB: Hemoglobin; RBC: Red Blood Cell; PLT: Platelet; INR: International Normalized Ratio; PT: Prothrombin Time; APTT: Activated Partial Thromboplastin Time; CREA: Creatinine; UREA: Urea; ALB: Albumin; TBIL: Total Bilirubin; DBIL: Direct Bilirubin; IBIL: Indirect Bilirubin; AST: Aspartate Aminotransferase; ALT: Alanine Aminotransferase; CRP: C-Reactive Protein; PCT: Procalcitonin; WBC: White Blood Cell; LAC: Lactate.

**Supplementary Table 15.** Characteristics of TXA group and Non-TXA group patients at ICU admission: Subgroup analysis of patients using PPI.

| Parameters (mean ± SD) | TXA group (n=58) | Non-TXA group (n=51) | P-value |
| --- | --- | --- | --- |
| Age, y | 54.47 ± 16.79 | 50.47 ± 16.23 | 0.211 |
| Male n% | 44 (75.9%) | 38 (74.5%) | >0.999 |
| APACHE Ⅱ | 21.36 ± 7.51 | 23.35 ± 7.55 | 0.171 |
| Type of admission |  |  | 0.201 |
| Medical n% | 39 (67.2%) | 29 (56.9%) |  |
| Surgical n% | 19 (32.8%) | 22 (43.1%) |  |
| Complication |  |  |  |
| Diabetes n% | 35 (14.3%) | 0 | 0.235 |
| CKD n% | 0 | 0 |  |
| Acute coronary syndrome n% | 2 (3.4%) | 6 (11.8%) | 0.196 |
| Severe pneumonia n% | 6 (28.6%) | 6 (33.3%) | >0.999 |
| Burn victim n% | 0 | 0 |  |
| Cancer n% | 5 (23.8%) | 2 (11.1%) | 0.418 |
| Neurological disorders n% | 6 (28.6%) | 5 (27.8%) | >0.999 |
| Severe acute pancreatitis n% | 3 (14.3%) | 3 (16.7%) | >0.999 |
| Trauma n% | 3 (14.3%) | 6 (33.3%) | 0.255 |

CKD: Chronic Kidney Disease.

**Supplementary Table 16.** Characteristics of TXA group and Non-TXA group patients on the day of 24-hour landmark after non-clinically important NVUGIB: Subgroup analysis of patients using PPI.

| Parameters (mean ± SD) / median (IQR) | TXA group (n=58) | Non-TXA group (n=51) | P-value |
| --- | --- | --- | --- |
| Time to Bleeding, d | 10 (0, 19) | 17 (6, 25) | 0.784 |
| SOFA-2 score | 6.14 ± 3.95 | 6.12 ± 4.71 | 0.981 |
| HGB, g/L | 88.97 ± 19.35 | 93.57 ± 18.11 | 0.202 |
| PLT, 10^9^/L | 191.29 ± 142.14 | 205.27 ± 146.48 | 0.615 |
| INR | 1.26 ± 0.42 | 1.24 ± 0.35 | 0.819 |
| APPT, sec | 34.90 ± 7.53 | 38.39 ± 22.91 | 0.302 |
| PT, sec | 14.38 ± 4.65 | 14.44 ± 3.67 | 0.939 |
| CREA, umol/L | 109.40 ± 95.28 | 115.39 ± 130.86 | 0.788 |
| UREA, umol/L | 13.26 ± 8.76 | 13.76 ± 11.94 | 0.648 |
| ALB, g/L | 34.35 ± 5.48 | 34.21 ± 5.28 | 0.891 |
| TBIL, umol/L | 40.69 ± 52.84 | 34.21 ± 62.87 | 0.564 |
| DBIL, umol/L | 30.38 ± 45.47 | 24.38 ± 50.60 | 0.519 |
| CRP, mg/L | 137.97 ± 128.71 | 87.10 ± 89.64 | 0.054 |
| PCT, ng/mL | 4.22 ± 9.11 | 3.01 ± 6.49 | 0.422 |
| WBC, 10^9^/L | 11.53 ± 6.55 | 11.83 ± 7.03 | 0.821 |
| LAC, mmol/L | 1.79 ± 1.15 | 1.81 ± 1.15 | 0.927 |
| Treatment |  |  |  |
| Vasoactive-inotropic drugs n% | 5 (23.8%) | 4 (22.2%) | >0.999 |
| Maximum infusion rate of norepinephrine, μg/kg/min | 0 (0, 0.13) | 0 (0, 0.20) | 0.138 |
| Mechanical ventilation n% | 9 (42.9%) | 12 (66.7%) | 0.244 |
| Proton pump inhibitors n% | 10 (47.6%) | 3 (16.7%) | 0.088 |
| Somatostatin n% | 6 (28.6%) | 4 (22.2%) | 0.932 |
| Anticoagulant drugs n% | 10 (17.2%) | 3 (5.9%) | 0.082 |
| Red blood cell suspension transfusion n% | 1 (4.8%) | 0 | >0.999 |
| Plasma transfusion n% | 3 (14.3%) | 1 (5.6%) | 0.609 |
| Enteral Nutrition n% | 11 (19.0%) | 10 (19.6%) | >0.999 |

Time to Bleeding: The number of days from admission to the ICU to the day of 24-hour landmark after non-clinically important NVUGIB. SOFA-2: Sequential Organ Failure Assessment-2; HGB: Hemoglobin; RBC: Red Blood Cell; PLT: Platelet; INR: International Normalized Ratio; PT: Prothrombin Time; APTT: Activated Partial Thromboplastin Time; CREA: Creatinine; UREA: Urea; ALB: Albumin; TBIL: Total Bilirubin; DBIL: Direct Bilirubin; IBIL: Indirect Bilirubin; AST: Aspartate Aminotransferase; ALT: Alanine Aminotransferase; CRP: C-Reactive Protein; PCT: Procalcitonin; WBC: White Blood Cell; LAC: Lactate.

**Supplementary Table 17.** Outcomes of patients in the TXA group and Non-TXA group: Subgroup analysis of patients with SOFA-2 score ≥ 6.

| Parameters (mean ± SD) / median (IQR) | TXA group (n=73) | Non-TXA group (n=64) | P-value |
| --- | --- | --- | --- |
| LOS, d | 30 (20, 44) | 25 (18, 43) | 0.514 |
| Mechanical Ventilation free in 28 days, d | 8 (1, 17) | 17 (6, 24) | 0.007 |
| Mechanical Ventilation time, d | 20 (11, 27) | 11 (4, 22) | 0.102 |
| RBC transfused post-bleeding n% | 6 (8.2%) | 5 (7.8%) | 0.930 |
| Days of UGIB within 28 days after 24-hour landmark, d | 3 (2, 5) | 3 (2, 4) | 0.054 |
| Endoscopy n% | 20 (27.4%) | 17 (26.6%) | >0.999 |

LOS: length of stay; GIB: Gastrointestinal Bleeding; RBC: Red blood cell.

**Supplementary Table 18.** Outcomes of patients in the TXA group and Non-TXA group: Subgroup analysis of patients with shock status.

| Parameters (mean ± SD) / median (IQR) | TXA group (n=71) | Non-TXA group (n=72) | P-value |
| --- | --- | --- | --- |
| LOS, d | 27 (16, 40) | 24 (16, 45) | 0.354 |
| Mechanical Ventilation free in 28 days, d | 14 (0, 22) | 17 (8, 24) | 0.053 |
| Mechanical Ventilation time, d | 14 (6, 28) | 11 (4, 20) | 0.401 |
| RBC transfused post-bleeding n% | 6 (8.5%) | 4 (5.6%) | 0.532 |
| Days of UGIB within 28 days after 24-hour landmark, d | 3 (2, 4) | 3 (2, 5) | 0.662 |
| Endoscopy n% | 16 (22.5%) | 22 (30.6%) | 0.370 |

LOS: length of stay; GIB: Gastrointestinal Bleeding; RBC: Red blood cell.

**Supplementary Table 19.** Outcomes of patients in the TXA group and Non-TXA group: Subgroup analysis of patients with sepsis.

| Parameters (mean ± SD) / median (IQR) | TXA group (n=88) | Non-TXA group (n=89) | P-value |
| --- | --- | --- | --- |
| LOS, d | 28 (17, 40) | 24 (16, 49) | 0.389 |
| Mechanical Ventilation free in 28 days, d | 12 (0, 20) | 16 (6, 24) | 0.088 |
| Mechanical Ventilation time, d | 16 (8, 27) | 12 (4, 22) | 0.988 |
| RBC transfused post-bleeding n% | 7 (8.0%) | 5 (5.6%) | 0.750 |
| Days of UGIB within 28 days after 24-hour landmark, d | 3 (2, 5) | 3 (1, 4) | 0.538 |
| Endoscopy n% | 24 (27.3%) | 18 (20.2%) | 0.355 |

LOS: length of stay; GIB: Gastrointestinal Bleeding; RBC: Red blood cell.

**Supplementary Table 20.** Outcomes of patients in the TXA group and Non-TXA group: Subgroup analysis of patients using anticoagulants.

| Parameters (mean ± SD) / median (IQR) | TXA group (n=21) | Non-TXA group (n=18) | P-value |
| --- | --- | --- | --- |
| LOS, d | 27 (17, 50) | 32 (17, 48) | 0.967 |
| Mechanical Ventilation free in 28 days, d | 11 (4, 21) | 10 (0, 20) | 0.544 |
| Mechanical Ventilation time, d | 17 (7, 24) | 17 (8, 32) | 0.959 |
| RBC transfused post-bleeding n% | 1 (4.8%) | 2 (11.1%) | 0.586 |
| Days of UGIB within 28 days after 24-hour landmark, d | 3 (2, 5) | 4 (1, 5) | 0.296 |
| Endoscopy n% | 5 (23.8%) | 4 (22.2%) | 0.288 |

LOS: length of stay; GIB: Gastrointestinal Bleeding; RBC: Red blood cell.

**Supplementary Table 21.** Outcomes of patients in the TXA group and Non-TXA group: Subgroup analysis of patients with a primary neurological diagnosis.

| Parameters (mean ± SD) / median (IQR) | TXA group (n=29) | Non-TXA group (n=36) | P-value |
| --- | --- | --- | --- |
| LOS, d | 31 (16, 50) | 29 (19, 48) | 0.301 |
| Mechanical Ventilation free in 28 days, d | 15 (5, 20) | 17 (6, 23) | 0.544 |
| Mechanical Ventilation time, d | 13 (8, 23) | 11 (5, 22) | 0.959 |
| RBC transfused post-bleeding n% | 1 (3.4%) | 1 (2.8%) | >0.999 |
| Days of UGIB within 28 days after 24-hour landmark, d | 3 (2, 4) | 3 (1, 4) | 0.296 |
| Endoscopy n% | 3 (10.3%) | 9 (25.0%) | 0.233 |

LOS: length of stay; GIB: Gastrointestinal Bleeding; RBC: Red blood cell.

**Supplementary Table 22.** Outcomes of patients in the TXA group and Non-TXA group: Subgroup analysis of patients with acute severe pancreatitis.

| Parameters (mean ± SD) / median (IQR) | TXA group (n=35) | Non-TXA group (n=29) | P-value |
| --- | --- | --- | --- |
| LOS, d | 35 (23, 43) | 23 (18, 34) | 0.150 |
| Mechanical Ventilation free in 28 days, d | 8 (6, 24) | 16 (8, 22) | 0.109 |
| Mechanical Ventilation time, d | 20 (4, 28) | 12 (5, 19) | 0.115 |
| RBC transfused post-bleeding n% | 4 (11.4%) | 1 (3.4%) | 0.366 |
| Days of UGIB within 28 days after 24-hour landmark, d | 3 (2, 5) | 3 (2, 5) | 0.829 |
| Endoscopy n% | 8 (22.9%) | 9 (31.0%) | 0.651 |

LOS: length of stay; GIB: Gastrointestinal Bleeding; RBC: Red blood cell.

**Supplementary Table 23.** Outcomes of patients in the TXA group and Non-TXA group: Subgroup analysis of patients using PPI.

| Parameters (mean ± SD) / median (IQR) | TXA group (n=58) | Non-TXA group (n=51) | P-value |
| --- | --- | --- | --- |
| LOS, d | 35 (24, 49) | 25 (18, 44) | 0.177 |
| Mechanical Ventilation free in 28 days, d | 10 (0, 19) | 17 (6, 25) | 0.015 |
| Mechanical Ventilation time, d | 18 (9, 28) | 11 (2, 22) | 0.075 |
| RBC transfused post-bleeding n% | 6 (10.3%) | 3 (5.9%) | 0.498 |
| Days of UGIB within 28 days after 24-hour landmark, d | 3 (2, 4) | 3 (2, 4) | 0.871 |
| Endoscopy n% | 18 (31.0%) | 8 (15.7%) | 0.099 |

LOS: length of stay; GIB: Gastrointestinal Bleeding; RBC: Red blood cell.
